# Cardiometabolic Health During the Climacteric Transition: A Narrative Review of Lifestyle, Physiological, and Nutritional Approaches

**DOI:** 10.3390/healthcare14121649

**Published:** 2026-06-10

**Authors:** María-Raquel Huerta-Franco, Solange Ivette Rivera-Manrique, Isabel Delgadillo-Holtfort, Svetlana Kashina, Carlos Eduardo Molina-Guerrero, José Marco Balleza-Ordaz, Francisco Miguel Vargas-Luna

**Affiliations:** 1 Departamento de Ciencias Aplicadas al Trabajo, División de Ciencias de la Salud, Campus León, Universidad de Guanajuato, León 37670, Guanajuato, Mexico; 2 Departamento de Ingenierías Química, Electrónica y Biomédica (DIQEB), División de Ciencias e Ingenierías, Campus León, Universidad de Guanajuato, León 37150, Guanajuato, Mexico; si.rivera@ugto.mx (S.I.R.-M.); ce.molina@ugto.mx (C.E.M.-G.); 3 Departamento de Ingeniería Física, División de Ciencias e Ingenierías, Campus León, Universidad de Guanajuato, León 37150, Guanajuato, Mexico; idelgadilloh@ugto.mx (I.D.-H.); k.svetlana@ugto.mx (S.K.); jm.balleza@ugto.mx (J.M.B.-O.); francisco.vargas@ugto.mx (F.M.V.-L.)

**Keywords:** climacteric transition, cardiometabolic health, visceral adiposity, sarcopenia, metabolic syndrome, heart rate variability, autonomic regulation, phytoestrogens, Mediterranean diet, exercise

## Abstract

**Highlights:**

**What are the main findings?**

- The climacteric transition is characterized by increased visceral adiposity, reduced lean mass, and a higher prevalence of cardiometabolic risk factors.
- Autonomic dysfunction, reflected by alterations in heart rate variability, together with lifestyle factors such as diet and exercise, plays a key role in midlife health.

**What are the implications of the main findings?**

- Integrative lifestyle interventions, including dietary strategies, phytoestrogens, and structured exercise, may improve cardiometabolic and autonomic health in climacteric women.
- Further longitudinal and interventional studies are needed to clarify mechanisms and develop personalized prevention strategies.

**Abstract:**

**Background/Objectives:** The climacteric transition is a critical stage in women’s health characterized by significant endocrine, metabolic, cardiovascular, and autonomic changes that increase cardiometabolic vulnerability during midlife. This narrative review aimed to synthesize current evidence on body composition, heart rate variability and autonomic function, phytoestrogens & estrobolome interactions, and exercise-based lifestyle approaches during the climacteric transition. **Methods:** A structured literature search was conducted across four domains (body composition, heart rate variability, phytoestrogens, and exercise) using PubMed/MEDLINE, Web of Science, Scopus, Google Scholar, and the Cochrane Library. Studies were selected based on relevance, study design, and methodological rigor, and synthesized using a narrative approach. Additional thematic components, including dietary patterns and gut microbiota estrobolome interactions, were incorporated through targeted searches. **Results:** The climacteric transition is associated with increased visceral adiposity, reduced lean mass, insulin resistance, dyslipidemia, and a higher prevalence of metabolic syndrome, while body mass index may underestimate metabolically relevant adiposity. Altered autonomic regulation, reflected by reduced heart rate variability and sympathetic predominance, is linked to increased cardiovascular risk, although its independent contribution is influenced by aging and comorbidities. Mediterranean and plant-based dietary patterns may improve metabolic and inflammatory profiles and modulate estrogen metabolism through gut microbiota mechanisms. Phytoestrogens show potential benefits for vasomotor symptoms and selected metabolic markers, although evidence remains heterogeneous. Exercise interventions consistently improve body composition, cardiometabolic parameters, and autonomic function. **Conclusions:** A multidimensional lifestyle-based approach integrating exercise, dietary strategies, and modulation of estrogen-related pathways may help mitigate cardiometabolic risk and support healthier aging during the climacteric transition.

## 1. Introduction

The climacteric transition represents a critical period in women’s health characterized by profound endocrine, metabolic, autonomic, and physiological changes. Although the decline in ovarian hormones, particularly estrogen and progesterone, has consistently been associated with increased cardiometabolic vulnerability, the mechanisms underlying individual variability in cardiometabolic outcomes remain incompletely understood [1,2,3,4]. Not all women experience the climacteric transition in the same manner; while some maintain relatively stable metabolic profiles, others develop central adiposity, insulin resistance, autonomic dysfunction, dyslipidemia, and elevated cardiovascular risk during midlife [5,6,7]. This heterogeneity suggests that cardiometabolic health during the climacteric transition cannot be explained solely by hormonal decline, but rather by the interaction of multiple interconnected physiological and lifestyle-related mechanisms.

Current evidence indicates that body composition changes, autonomic function and heart rate variability, sleep-related metabolic regulation, dietary patterns, phytoestrogen exposure, gut microbiota-related pathways, and physical activity may collectively influence cardiometabolic trajectories during the menopausal transition [8,9,10,11,12]. However, these domains are frequently studied in isolation, resulting in a fragmented understanding of how metabolic, neuroendocrine, autonomic, and lifestyle-related factors interact to influence cardiometabolic vulnerability and health outcomes in midlife women. Consequently, despite the growing volume of evidence, important gaps remain regarding the integration of these mechanisms into a clinically relevant understanding of cardiometabolic health during the climacteric transition. Addressing this limitation may help support a more integrated perspective on the physiological and lifestyle-related factors associated with cardiometabolic vulnerability in midlife women.

Beyond changes in body composition and metabolic health, alterations in autonomic regulation and sleep patterns have also emerged as relevant features of the climacteric transition [10,11,12,13]. Vasomotor symptoms, sleep disturbances, and reduced heart rate variability have been associated with complex interactions among endocrine changes, autonomic regulation, hypothalamic–pituitary–adrenal axis activity, and stress-related physiological responses [9,10,11,12,13]. Heart rate variability, a widely used marker of autonomic nervous system function, has gained increasing attention as a potential indicator of cardiovascular adaptability and physiological resilience during midlife [10,11,12]. Furthermore, impaired sleep quality and circadian disruption have been associated with adverse cardiometabolic outcomes, including weight gain, hypertension, impaired glucose regulation, systemic inflammation, and reduced quality of life [12,13]. Together, these observations highlight autonomic regulation and sleep-related processes as increasingly important determinants of cardiometabolic health during the menopausal transition.

A hallmark of the menopausal transition is the redistribution of body fat toward a central or visceral pattern, occurring partly independent of chronological aging and closely associated with hormonal decline [5,6,7]. In parallel, progressive reductions in lean body mass and skeletal muscle function may contribute to early manifestations of sarcopenia and an increased risk of sarcopenic obesity in postmenopausal women [5,6,7]. These alterations in body composition have been associated with insulin resistance, dyslipidemia, endothelial dysfunction, chronic low-grade inflammation, and an increased prevalence of metabolic syndrome after menopause [5,6,7,8,9,10]. Collectively, these findings highlight body composition changes as a key link between endocrine aging and cardiometabolic risk during midlife (Table 1).

Acute stress exposure has been associated with altered gastric motility responses in peri- and postmenopausal women, suggesting potential alterations in autonomic and neurophysiological regulation during the menopausal transition [14]. In parallel, perceived stress, systemic arterial hypertension, type 2 diabetes mellitus, and elevated fasting glucose have been identified as independent predictors of metabolic syndrome [15]. These observations suggest that stress-related physiological responses may interact with autonomic regulation and metabolic dysfunction, contributing to cardiometabolic risk during midlife [15,16].

In this context, heart rate variability (HRV) has emerged as a valuable noninvasive marker of autonomic nervous system function and cardiovascular regulation during the climacteric transition [10,11,14]. Reduced HRV has been associated with sympathetic predominance, altered autonomic modulation, and diminished cardiovascular adaptability in midlife women [10,11,14]. In addition, sleep disturbances and circadian disruption, which are common during menopause, may further influence autonomic regulation and cardiometabolic health [12,13]. Together, these findings support the inclusion of HRV as a relevant physiological indicator linking autonomic regulation with cardiometabolic health during the menopausal transition.

**Figure 1:**
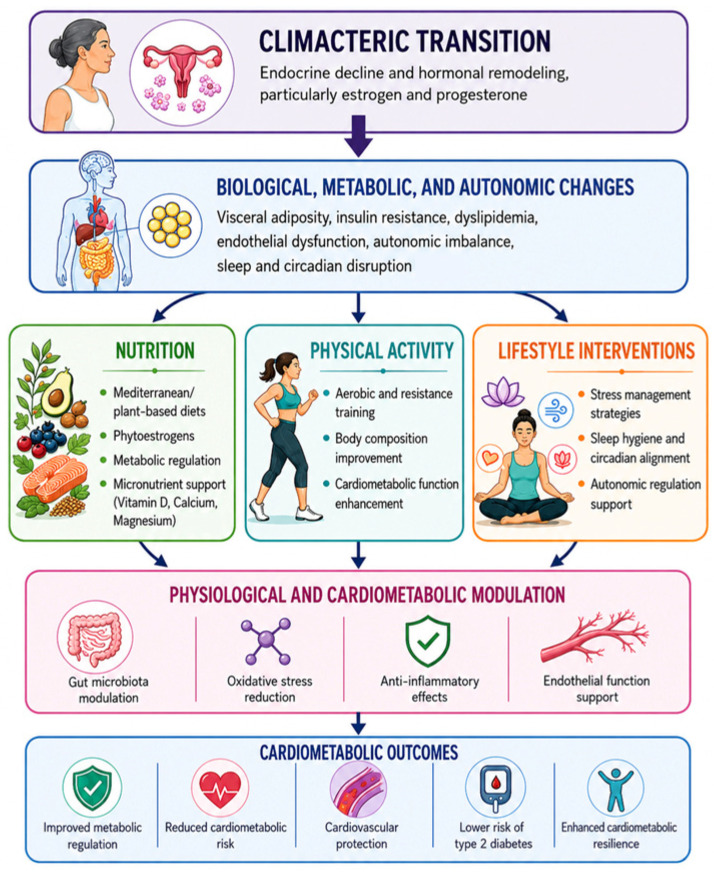
Conceptual framework of cardiometabolic health during the climacteric transition. Endocrine changes associated with ovarian aging influence body composition, metabolic regulation, autonomic function, vascular health, and sleep–circadian homeostasis [1,5,10,12]. These physiological adaptations may contribute to cardiometabolic risk, whereas modifiable lifestyle factors (including dietary patterns, phytoestrogen intake, physical activity, and healthy sleep behaviors) may support pathways associated with cardiometabolic health during midlife [1,2,3,4,5,6,7,8,9,10,11,12,13,16,17,18,19,20].

Lifestyle-based strategies have emerged as promising approaches to mitigate cardiometabolic vulnerability during the climacteric transition. Healthy dietary practices and other lifestyle modifications have been recommended as part of comprehensive preventive strategies for postmenopausal women, with potential benefits extending beyond bone health to overall metabolic and cardiovascular well-being [17]. Likewise, aerobic and resistance exercise have been associated with improvements in physical fitness, metabolic health, body composition, and overall quality of life in women during the climacteric transition [18]. Mind–body interventions such as yoga have also demonstrated benefits for psychological symptoms, including anxiety, mood disturbances, and sleep-related complaints, supporting their potential role in promoting well-being during menopause [19]. Together, these findings support the potential role of lifestyle and behavioral interventions in promoting cardiometabolic health and healthy aging during the menopausal transition. Additionally, recent evidence suggests that reducing sedentary behavior and increasing sit-to-stand transitions may represent feasible behavioral strategies to improve cardiovascular health in postmenopausal women [20] (Figure 1).

Therefore, this narrative review examines current evidence on cardiometabolic health during the climacteric transition, focusing on four interrelated domains: body composition, heart rate variability, phytoestrogens and estrobolome, and exercise interventions. These domains represent key physiological and modifiable factors associated with metabolic regulation, cardiovascular function, and overall health in midlife women. By integrating evidence across these areas, this review aims to provide a comprehensive, multidimensional, and mechanistic perspective on cardiometabolic health during the climacteric transition.

## 2. Materials and Methods

### 2.1. Review Design

This integrative review was organized into four thematic domains relevant to cardiometabolic health during the climacteric transition: (1) body composition and cardiometabolic risk, (2) heart rate variability as an indicator of autonomic function, (3) phytoestrogens and estrobolome-related mechanisms, and (4) physical exercise interventions. These domains encompass key physiological and modifiable factors associated with metabolic regulation, cardiovascular function, and adaptive responses to endocrine changes during midlife.

Given the interdisciplinary nature of cardiometabolic health during the climacteric transition, evidence was organized according to thematic relevance within each domain. Findings from the four domains were critically examined and synthesized to provide a broader understanding of the physiological and lifestyle-related factors associated with cardiometabolic health in midlife women.

### 2.2. Search Strategy and Data Sources

Relevant literature was identified through searches conducted in Web of Science Core Collection, Scopus, PubMed/MEDLINE, PubMed Central (PMC), and the Cochrane Library. Google Scholar was additionally consulted for selected domains, particularly those related to phytoestrogens, estrobolome-related mechanisms, and physical exercise interventions. The search focused primarily on literature published between January 2020 and December 2026, with emphasis on recent evidence relevant to cardiometabolic health during the climacteric transition.

Representative literature searches were guided by terms related to menopause and the climacteric transition, body composition, cardiometabolic risk, autonomic function and heart rate variability, phytoestrogens and estrobolome-related mechanisms, and physical exercise. Examples of search terms included “menopause,” “climacteric,” “body composition,” “visceral adiposity,” “sarcopenia,” “metabolic syndrome,” “cardiometabolic risk,” “phytoestrogens,” “estrobolome,” “heart rate variability,” “exercise,” and “physical activity.” Boolean operators (AND/OR) were adapted according to the syntax and requirements of each database.

The review focused primarily on peer-reviewed studies published within the last seven years, while seminal and highly cited publications were included when relevant to provide historical and conceptual context for the thematic domains examined.

### 2.3. Thematic Scope

The review focused on studies involving women undergoing the menopausal transition, climacteric, perimenopause, or postmenopause. Evidence was examined in relation to the four thematic domains of interest, including body composition and adiposity-related measures, autonomic and cardiovascular indicators assessed through heart rate variability, phytoestrogen and estrobolome-related mechanisms, and outcomes associated with physical exercise interventions. Additional variables considered within these domains included vasomotor symptoms, metabolic syndrome, bone health, lipid profile, oxidative stress, inflammatory markers, and other factors relevant to cardiometabolic health during midlife.

The evidence considered in this review included original studies conducted in human populations, such as observational studies and randomized controlled trials across the four thematic domains. Review articles were also consulted selectively when they provided relevant conceptual, mechanistic, or contextual information that supported the interpretation of the available evidence.

Sources not considered in the present review included studies conducted exclusively in non-human models, investigations involving only male populations, and publications not directly related to the thematic focus of the review. Editorials, conference abstracts, and other non-peer-reviewed sources were also not considered.

### 2.4. Literature Identification and Thematic Organization

**Figure 2:**
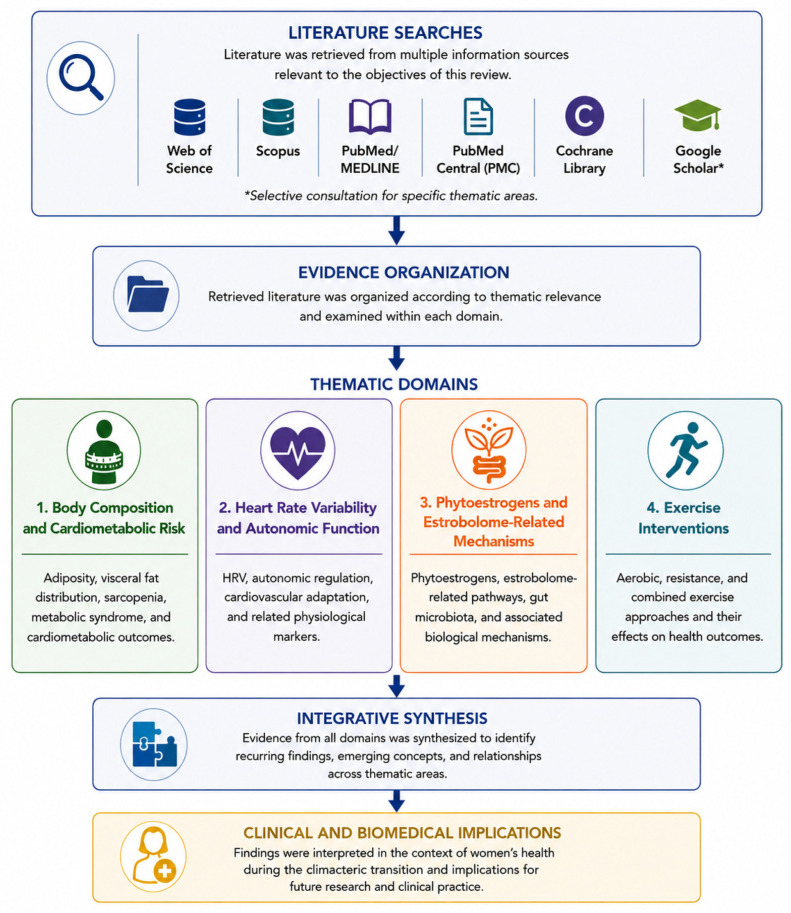
Schematic representation of the integrative review framework. Literature retrieved from multiple information sources was organized according to thematic relevance and synthesized to facilitate an integrated understanding of factors associated with cardiometabolic health during the climacteric transition. * Google Scholar was selectively consulted for specific thematic areas, particularly phytoestrogens, estrobolome-related mechanisms, and exercise interventions.

Relevant literature was identified and organized according to thematic relevance. Evidence from each domain was examined to identify recurring findings, emerging concepts, and relationships relevant to cardiometabolic health during the climacteric transition. A schematic overview of this approach is presented in Figure 2.

### 2.5. Literature Organization and Narrative Synthesis

Information from the reviewed literature was organized according to the objectives of each thematic domain. Data related to study characteristics, participant profiles, menopausal stage, interventions or exposures, and principal findings were examined and synthesized narratively. This process facilitated the identification of recurring findings, emerging concepts, and clinically relevant relationships across literature. A schematic overview of the evidence organization and synthesis process is presented in Figure 2.

### 2.6. Methodological Considerations

The evidence base included observational studies, interventional studies, and selected review articles that provided complementary conceptual and mechanistic perspectives. Given the diversity of study designs, populations, interventions, and outcome measures, the reviewed literature was critically appraised through the examination of study characteristics, methodological approaches, and principal findings. Attention was given to the consistency of reported results, areas of agreement and divergence across studies, and the relevance of the evidence to the thematic objectives of the review. This interpretative approach facilitated the integration of evidence from multiple domains and supported a comprehensive understanding of factors associated with cardiometabolic health during the climacteric transition.

## 3. Results

### 3.1. Body Composition Changes and Cardiometabolic Risk During the Climacteric Transition

#### 3.1.1. Sarcopenia and Sarcopenic Obesity as Key Components of Body Composition Changes During the Climacteric Transition

The characteristics of the 31 studies included in the qualitative synthesis are summarized in Table 2 [21,22,23,24,25,26,27,28,29,30,31,32,33,34,35,36,37,38,39,40,41,42,43,44,45,46,47,48,49,50,51]. Overall, the available evidence indicates that the climacteric transition is accompanied by substantial alterations in body composition, characterized by progressive reductions in lean mass and skeletal muscle function together with increased visceral adiposity [21,22,23,24,28]. These changes may contribute to the development of sarcopenia and sarcopenic obesity, conditions increasingly recognized as important contributors to cardiometabolic vulnerability in midlife and postmenopausal women [25,26,31,40]. Furthermore, visceral fat accumulation has been consistently associated with metabolic syndrome, insulin resistance, and increased cardiometabolic risk across multiple populations, although the strength of these associations appears to vary according to study design, population characteristics, and the adiposity indices employed [23,24,31,40].

Table 2—Summary of studies examining body composition changes during the climacteric transition and their reported associations with cardiometabolic health. Included studies evaluated parameters related to visceral adiposity, fat distribution, lean mass, sarcopenia, and sarcopenic obesity in premenopausal (PRE), perimenopausal (PERI), and postmenopausal (PM) women. Narrative reviews and meta-analyses are identified in the study design column and were included to provide contextual and integrative perspectives alongside primary empirical studies.

In addition, surrogate markers of visceral adiposity, including the Visceral Adiposity Index (VAI) and Lipid Accumulation Product (LAP), have emerged as useful indicators associated with atherogenic and cardiovascular risk profiles in postmenopausal women, highlighting the clinical relevance of visceral adipose dysfunction beyond conventional anthropometric measures [32]. In Venezuelan postmenopausal women, increasing adiposity, particularly abdominal obesity and elevated waist-to-height ratio, was associated with higher triglyceride concentrations, lower HDL-cholesterol levels, and a more adverse cardiometabolic profile, reflected by elevated TG/HDL and TC/HDL ratios [33]. Together, these findings support the potential value of visceral adiposity-related indices for identifying women with unfavorable cardiometabolic profiles during postmenopause [32,33].

In a large cohort of 705 postmenopausal women, visceral adiposity index (VAI) was independently associated with metabolic syndrome (OR = 2.07, 95% CI 1.73–2.48), while menopause duration greater than 5 years was strongly associated with a higher likelihood of metabolic syndrome (OR = 6.44, 95% CI 3.34–12.45), highlighting the potential contribution of central adiposity and menopausal duration to cardiometabolic risk [34]. Evidence from a recent systematic review and meta-analysis indicates that postmenopausal women exhibit greater adiposity, including higher BMI, waist circumference, and waist-to-hip ratio, compared with premenopausal women [36]. The consistency of these findings across observational studies and meta-analytic evidence underscores the relevance of adiposity-related changes during the menopausal transition [34,36].

Beyond anthropometric and adiposity-related indices, emerging evidence suggests that adipokine profiles may provide additional insight into metabolic dysfunction during the menopausal transition. In a cross-sectional study including peri- and postmenopausal women, lower adiponectin concentrations were associated with higher visceral adiposity indices, insulin resistance, adverse glycemic markers, and triglyceride levels, whereas positive associations were observed with HDL-cholesterol. These findings support adiponectin as a potential biomarker linking visceral adiposity, metabolic dysfunction, and cardiometabolic risk in midlife women [37].

In addition to cross-sectional indices of adiposity, longitudinal patterns of body weight change may provide further insight into cardiometabolic health during postmenopausal. Analysis of NHANES data demonstrated that postmenopausal women with increasing or increasing–decreasing BMI trajectories had approximately fivefold higher odds of metabolic syndrome compared with women who maintained a stable BMI over time, suggesting that long-term changes in body weight may be relevant to cardiometabolic risk during the menopausal transition [39].

Importantly, a multicenter DXA-based analysis demonstrated that up to 22% of women with a normal body mass index (<23 kg/m^2^) exhibited elevated visceral adiposity (VAT > 100 cm^2^), while 35.7% presented an android fat distribution pattern (A/G ratio > 1.0), indicative of central obesity. These findings suggest that BMI alone may not adequately identify women with unfavorable fat distribution patterns and potential cardiometabolic risk, particularly during the peri- and postmenopausal stages [41].

Anthropometric indices such as the Body Roundness Index (BRI) demonstrate moderate predictive value for metabolic syndrome (AUC = 0.75; 95% CI: 0.71–0.80), with an associated OR of 2.65 (95% CI: 1.99–3.53), sensitivity of 85.6%, and specificity of 72.5% at an optimal cut-off of 8.15, supporting its utility as a simple marker of cardiometabolic risk in postmenopausal women [42].

In parallel with these adiposity-related changes, progressive declines in lean body mass and skeletal muscle mass have been observed across menopausal stages, accompanied by increases in visceral fat area (from 36.4 to 55.7 cm^2^ from premenopause to postmenopause), reflecting a shift toward central adiposity and a less favorable body composition profile [25,26,44].

Increased adiposity has been consistently associated with elevated insulin resistance, higher circulating insulin levels, and increased concentrations of pro-inflammatory cytokines such as TNF-α and IL-6, reinforcing the role of chronic low-grade inflammation in cardiometabolic dysfunction [38,39]. In addition, alterations in iron metabolism have been implicated in this process. Matta et al. [27] reported that elevated hepcidin levels are associated with increased insulin resistance and visceral adiposity in pre- and postmenopausal women, indicating a possible link between iron homeostasis and metabolic dysfunction [27].

Collectively, these findings indicate that concurrent reductions in skeletal muscle mass and increases in adiposity are common features of body composition changes during the menopausal transition and are associated with unfavorable metabolic profiles [25,26,38,39,44].

Observational studies indicate that increased visceral adiposity is associated with lower muscle strength and reduced functional capacity, with handgrip strength emerging as a simple and reliable clinical marker of metabolic vulnerability [28]. From a metabolic perspective, skeletal muscle plays a central role in glucose uptake and insulin sensitivity; therefore, reductions in muscle mass may contribute to dysglycemia, dyslipidemia, and systemic inflammation, contributing to the progression of cardiometabolic disorders [28,38]. These findings indicate that alterations in body composition during the climacteric transition extend beyond simple weight gain and involve complex interactions between adiposity and muscle loss, which are not adequately captured by conventional anthropometric measures (Table 2). Accordingly, these observations emphasize the importance of assessing body composition beyond BMI by incorporating measures of fat distribution, lean mass, and muscle function to improve cardiometabolic risk stratification.

Furthermore, intervention studies have shown that lifestyle-based approaches may improve body composition and selected cardiometabolic parameters in postmenopausal women [45]. Similarly, a long-term yoga intervention in sedentary climacteric women with metabolic syndrome was associated with a significant reduction in MetS prevalence (up to 46% at 24 months), accompanied by improvements in fasting glucose, HDL-cholesterol, waist circumference, and blood pressure, indicating favorable changes in cardiometabolic parameters associated with metabolic syndrome [47].

Further evidence supports the benefits of multimodal lifestyle interventions. Combining exercise with time-restricted eating (16:8) produced greater improvements in body composition and cardiometabolic health than exercise alone. Significant reductions were observed in BMI, fat mass, glucose, insulin, HOMA-IR, waist circumference, and waist-to-height ratio, indicating concurrent improvements in adiposity-related and metabolic parameters in menopausal women [48].

Dietary factors may also contribute to cardiometabolic health during the climacteric transition. Greater adherence to the Mediterranean diet was associated with lower odds of central obesity and hypertension among postmenopausal women, although no significant association was observed with metabolic syndrome overall [50].

Lifestyle interventions, particularly resistance training combined with adequate protein intake, have been associated with beneficial effects on muscle mass, strength, and metabolic outcomes. Resistance training interventions in postmenopausal women have been shown to increase lean mass, improve muscle strength and quality, reduce regional fat mass, and enhance glucose tolerance [45]. Furthermore, regular physical activity has been associated with reductions in visceral adiposity and improvements in insulin sensitivity, supporting its potential role in promoting cardiometabolic health during the menopausal transition [47,48]. Overall, sarcopenic obesity represents an important body composition phenotype during the climacteric transition and has been associated with adverse metabolic and functional outcomes in midlife women. The available evidence highlights the potential relevance of preserving both muscle mass and metabolic health during the menopausal transition.

#### 3.1.2. Key Insights on Sarcopenia and Sarcopenic Obesity

Overall, the available evidence supports a broader understanding of cardiometabolic health during the menopausal transition, moving beyond a static, hormone-centered model toward a dynamic, integrative, and life-course–based framework. Collectively, the findings reviewed suggest that cardiometabolic vulnerability is influenced by the interaction among visceral adiposity, skeletal muscle decline, hormonal changes, metabolic regulation, and long-term patterns of weight change. This integrated perspective highlights the importance of evaluating body composition beyond BMI and considering multiple dimensions of risk when assessing cardiometabolic health in midlife women.

### 3.2. Heart Rate Variability, Autonomic Regulation, and Cardiometabolic Health During the Climacteric Transition

#### Heart Rate Variability as a Marker of Autonomic and Cardiometabolic Health

Heart rate variability (HRV), which is tightly regulated by the autonomic nervous system (ANS), has been used for decades as a non-invasive tool to assess cardiovascular health. It reflects the dynamic balance between sympathetic and parasympathetic (vagal) activity. Lower HRV generally indicates reduced autonomic flexibility and is associated with increased cardiovascular risk. HRV is quantified through time-domain, frequency-domain, and nonlinear indices derived from the variation in successive normal-to-normal (NN) RR intervals on the electrocardiogram [52].

In menopausal women, HRV is commonly used to evaluate autonomic cardiovascular changes associated with hormonal shifts. Several studies have documented a decline in HRV and a shift toward sympathetic dominance in PM women [53,54]. In fact, in a 2016 review, von Holzen et al. [55] concluded that all included investigations agreed on a postmenopausal decline in HRV accompanied by a shift toward higher sympathetic control.

However, because aging itself exerts a strong influence on cardiovascular parameters, the evidence is not uniform. Isolating the specific impact of menopause remains challenging. Carvalho et al. [56] evaluated 17 HRV indices (time-domain, frequency-domain, geometric, and nonlinear) for their ability to predict late PM status. They found only weak but significant negative correlations with time since menopause, with the geometric index RRtri showing the strongest predictive value. Similarly, Ramesh et al. [57] concluded that age, rather than endogenous estradiol levels, is the primary driver of reduced HRV, although menopausal status was still associated with impaired HRV responses to stress. Solanki et al. [58] reported no significant differences in 5 min HRV parameters among age-matched PRE-, PERI-, and PM women, further supporting the dominant role of chronological age.

Other studies have evaluated the effects of interventions on HRV exclusively in menopausal or PM women, some of them without including PRE or young women or men as control groups. Consequently, these designs cannot clearly determine whether the observed consequences of the interventions are specifically modulated by the menopausal status. For example, the relationship between physical activity/exercise and HRV in menopausal women has been examined in multiple studies. Rezende Barbosa et al. [59] assessed the impact of functional training on geometric indices and fractal correlation properties of HRV in PM women. Similarly, Sakai et al. [60] examined the effects of autogenic training on skin properties and cardiac autonomic activity via HRV in PM women only. Putra et al. [61] demonstrated improvements in SDNN and other HRV parameters following short-term combined exercise training in PM women. Praveena et al. [62] showed that 3 months of yoga practice significantly improved parasympathetic HRV indices in early PM women, suggesting potential cardiovascular protective effects. While all these studies proved benefits within PM cohorts, the absence of non-menopausal comparators limits conclusions regarding menopause-specific interactions with the interventions.

Some studies have included control groups. Regarding aerobic capacity, Thakkar et al. [53] found no correlation between HRV and aerobic capacity but confirmed an overall decline in SDNN with advancing menopause, “thereby aging” (textual quote).

Ongoing research continues to examine the stages of menopause while accounting for additional factors such as comorbidities or conditions commonly associated with aging and hormonal changes as, e.g., sleep disorders, and incorporating a broader range of HRV indices. For instance, Virtanen et al. [63] investigated the effects of sleep disturbance a condition known to increase sympathetic activity and highly prevalent during the climacteric- on overnight HRV in PERI- and PM women. They found only minor effects of sleep disturbance on HRV parameters, with no significant changes attributable to menopausal hormone therapy (MHT). In an earlier study, Virtanen et al. [64] examined the impact of 40 h of total sleep deprivation in PM and young women, focusing on age in the discussion, and reported deleterious effects on cardiac autonomic function across (time-domain, frequency-domain, and nonlinear indices) in women overall. However, these negative effects were more pronounced in PM women, and hormone therapy did not appear to offer protection. Similarly, De Zambotti et al. [65] studied women in the early menopausal transition with and without insomnia disorder compared with age-matched controls. They observed nocturnal autonomic hyperarousal in the insomnia group, evidenced by higher heart rate and a tendency toward lower high-frequency (HF) power, suggesting reduced vagal tone during both follicular and luteal phases.

Regarding menopause-specific symptoms such as Vasomotor Symptoms (VMS), Sánchez-Barajas et al. [66] investigated the associations between HRV and carotid artery stiffness, elasticity, and impedance in PERI and PM women. They observed higher SDNN in PM compared with PERI women but found no clear association between HRV and VMS. Multivariate analysis revealed significant relationships between indices of early carotid damage and several HRV parameters, interpreted as evidence of autonomic imbalance and increased cardiovascular disease risk. In a follow-up study, the same group evaluated carotid intima-media thickness (IMT) and flow-mediated dilatation (FMD) alongside HRV [67]. Late PM women showed greater carotid IMT and reduced FMD. HRV analysis indicated higher pNN50 in PRE women, elevated low-frequency (LF) power in both PM groups, and higher high-frequency (HF) power in the early PM group.

The relationship between autonomic function and VMS remains complex. Jones et al. [68] found no association between HRV parameters and the frequency or intensity of VMS in PERI and PM women, even among those participating in yoga or omega-3 supplementation interventions. In contrast, Stokes et al. [69] reported that PM women with VMS had lower resting heart rate and higher SDNN compared with those without VMS. Martinelli et al. [70] observed significantly lower vagal indices (RMSSD, pNN50, and HF power) in women with moderate-to-severe menopausal symptoms versus mild symptoms, with an inverse association between symptom intensity and RMSSD/pNN50. Similarly, Sahu et al. [71] reported decreased SDRR, RMSSD, pRR50, VLF, and HF, along with increased LF and LF/HF ratio in PM versus PRE women, with a further sympathetic predominance in symptomatic PM women.

As menopause involves significant hormonal changes, the study of MHT is particularly relevant. Von Holzen et al. [55] reported a generally supportive effect of estrogen on HRV (increased vagal modulation), although this benefit was typically abolished when progestogens were added in combined regimens. These findings contrast with those of Virtanen et al. [63], who observed no detectable effect of MHT on overnight HRV parameters in PERI- and PM women with sleep disturbance.

Other very common age-related comorbidities have also been examined. Philbois et al. [72] showed that PRE hypertensive women already exhibit impaired cardiac autonomic modulation, with lower HRV total power (TP) compared to normotensive PRE women, yet they maintain vagal predominance. In contrast, both normotensive and hypertensive PM women display a clear shift toward sympathetic predominance, which may partly be related to aging. However, hypertensive PM women exhibited even greater autonomic impairment than their normotensive PM counterparts.

In breast cancer survivors, Renna et al. [73] found that older age was significantly associated with lower HRV. However, HRV was not associated with menopausal status. These results further support the notion that chronological age may exert a stronger influence on HRV than menopausal hormonal changes in this population.

Diabetes prevalence is increasing worldwide. Type 1 diabetes is detected frequently in youth and young adults, but type 2 diabetes prevalence has a strong correlation with age. Therefore, diabetes and menopause combined have also been studied. Nattero-Chávez et al. [74] examined sex differences in cardiovascular autonomic neuropathy (CAN) in patients with type 1 diabetes. Using an age cutoff of 50 years (approximating the median age of natural menopause), they observed lower low-frequency (LF) and high-frequency (HF) power in patients older than 50 years in both sexes, with a more pronounced reduction in women. This contributed to a significantly higher prevalence of asymptomatic cardioautonomic neuropathy (CAN) in women over 50 years (or after menopause), an age-related excess risk that was not observed in men. In a related study with type 2 diabetes, Pervaiz et al. [75] compared PRE- and PM women and found that QTc interval prolongation was markedly higher in the PM group. They concluded that QTc prolongation may serve as an early predictor of CAN in PM diabetic women, often appearing before symptoms or other autonomic markers. Notably, no significant differences were detected in HRV parameters, resting heart rate, or Valsalva ratio among the groups.

The declination of cognitive abilities is another factor commonly associated with age and therefore investigated in older (menopause) women. Haldar et al. [76] investigated cognitive function and HRV in reproductive-age and PM women. They found that PM women exhibited significantly lower cognitive performance (MoCA scores) compared to reproductive-age women, along with higher parasympathetic HRV indices (RMSSD, pNN50, and HF power) and lower LF power. In a related review, Duval and Ditto [77] synthesized evidence of reduced cognitive performance in PM women relative to PRE and PERI stages. However, they noted that no studies have unequivocally examined the interaction between HRV and cognitive function across the adult female lifespan, highlighting an important research gap.

Almeida Júnior et al. [78] examined the impact of dry eye syndrome (DES) on cardiac autonomic modulation in PM women. This condition is influenced by the ANS and its prevalence significantly increases with menopause. The authors found no significant differences in HRV parameters between PM women with and without DES. Additionally, neither time since menopause nor the intensity of menopausal symptoms showed any association with HRV indices.

Daily activities and physiological challenges also affect HRV. Postural changes (from supine (−) to orthostatic (Δ) position) have been investigated in menopausal women. Scatà et al. [79] evaluated the cardiac autonomic response to an active standing test in four groups: young women, young men, older men, and PM women. They analyzed Δ0V% and ΔLFn as markers of sympathetic modulation, and Δ2UV% and ΔHFn as markers of vagal modulation. PM women exhibited a blunted autonomic response to orthostatic stress compared to both young groups, with a particularly pronounced reduction in sympathetic modulation (Δ0V% and ΔLFn) compared to all other groups (including older men). In an earlier study, Kangas et al. [80] assessed hemodynamic and HRV responses to passive head-up tilt in men and women. In participants older than 55 years, men showed higher LF/HF ratios (indicating greater sympathetic predominance) in both supine and upright positions compared to women.

Sexual activity appears to be a relevant cardioprotective factor in PM women. Tolunay et al. [81] reported that sexually active PM women exhibited significantly higher values across multiple HRV indices (both time- and frequency-domain) compared to sexually inactive women. Moreover, regression analysis showed that some indices (particularly SDNNI, RMSSD, and LF) were independently and positively associated with the weekly frequency of sexual activity.

While comparing PM women with PRE or young women is important, comparing them with groups of men is also relevant, despite the intrinsic hormonal differences between the sexes. Voss et al. [82] compared HRV indices between young and old groups of men and women. They reported gender differences in younger ages (higher sympathetic activity and lower parasympathetic tone in men, and the opposite in women) presumably due to differing hormonal profiles. However, these gender differences largely disappear with aging as hormonal changes (particularly menopause in women) lead to more comparable autonomic profiles between the sexes.

Table 3 summarizes evidence on autonomic function and HRV in climacteric women, showing that HRV decline is mainly driven by aging and is characterized by reduced vagal activity and increased sympathetic predominance in postmenopause. Additionally, factors such as sleep disturbances and comorbidities negatively affect autonomic regulation, while exercise-based interventions may improve HRV parameters.

### 3.3. Phytoestrogens and Nutritional Approaches

#### 3.3.1. Effects of Phytoestrogens (Isoflavones, Lignans) on Vasomotor Symptoms and Metabolism

Perimenopause is a complex transitional phase that varies substantially among women. Vasomotor symptoms (VMS) and metabolic manifestations are common features associated with altered thermoregulatory function during the menopausal transition. As noted by [83], some women experience this period as relatively benign, marked only by subtle physiological changes, while others experience disruptive physical and psychological symptoms that negatively affect quality of life.

Hormone therapies are the most effective treatments for perimenopausal symptoms, reducing symptom severity by more than 75%. Hormone therapy typically involves the administration of estrogens combined with progesterone to ensure endometrial protection. Estrogens may be delivered as 17*β* estradiol (via transdermal patches, gels, or sprays) or as oral conjugated estrogens [84]. Many women benefit from multi targeted approaches that address sleep disturbances, mood alterations, and genitourinary symptoms.

Although hormone therapy (either estrogen alone in women without a uterus or estrogen combined with a progestogen) effectively reduces VMS, it may increase the risk of coronary heart disease in older women and may increase breast cancer risk. Nonhormonal alternatives such as paroxetine mesylate (7.5 mg daily) significantly reduce VMS, sleep disturbances, and depressive or anxiety symptoms. Fezolinetant (45 mg daily) has also demonstrated efficacy in decreasing VMS, although it is frequently associated with headaches and nausea. Additional nonhormonal options, such as SSRIs and SNRIs, decrease VMS and improve sleep and mood; however, they may lead to weight gain, gastrointestinal adverse effects, and sexual dysfunction [84,85]. Other pharmacological treatments (including oxybutynin and gabapentin) exhibit moderate efficacy but can produce adverse effects such as urinary retention, constipation, dizziness, drowsiness, impaired balance, and potential cognitive decline with long term use. Levonorgestrel releasing intrauterine devices are commonly employed for contraceptive purposes or endometrial protection but may cause acne, abdominal pain, pelvic discomfort, amenorrhea, or headaches [84].

In this context, alternative treatments involving phytoestrogens have gained increasing scientific and clinical interest. Phytoestrogens are compounds with affinity for estro-gen receptors. These include isoflavones, coumestans, prenylated flavonoids, lignans, ellagitannins, and stilbenes (phenolic compounds naturally present in soybeans, hops, flaxseed, pomegranate, broccoli, and wine). Due to their structural similarity to human estrogens, these molecules may help reduce perimenopausal symptoms. The physiological effects of phytoestrogens are largely mediated by the intestinal microbiota, which converts dietary precursors into bioactive metabolites such as equol, coumestrol, 8-prenylnaringenin, enterolignans, urolithins, and resveratrol [86,87]. These microbial metabolites exhibit diverse estrogenic, antioxidant, and anti-inflammatory functions, positioning phytoestrogens as promising complementary strategies for symptom management during the menopausal transition.

According to [88], various therapies are used to reduce vasomotor symptoms (VMS), with phytoestrogen consumption being the fourth most reported treatment option among women experiencing menopause-related symptoms. Phytoestrogens, particularly soy derived isoflavones and lignans, have attracted growing interest as complementary nutritional approaches due to their potential beneficial effects on menopausal symptoms and cardiometabolic health. Several studies suggest that these compounds may contribute to improvements in lipid metabolism, vascular function, and overall quality of life during the menopausal transition.

Genistein and daidzein are isoflavones that are commonly found in soybeans [87]. Genistein is one of the most extensively studied phytoestrogens and is characterized by weak estrogenic activity mediated through estrogen receptor binding, as well as notable antioxidant and anti-inflammatory properties. It has been shown to exert beneficial effects on bone metabolism, contribute to the alleviation of menopausal symptoms, and be associated with the prevention of certain hormone-dependent cancers. Daidzein is an isoflavone that can be metabolized by intestinal microbiota into equol, a biologically more active metabolite, although this conversion occurs only in a subset of individuals. Daidzein and its metabolites have been associated with cardiovascular benefits, modulation of hormonal balance, and a reduction in menopausal symptoms [87].

Recent clinical evidence indicates that formulations containing soy isoflavones and lignans may help alleviate menopausal symptoms and are generally associated with good safety and tolerability profiles [89].

#### 3.3.2. Therapeutic Potential and Current Limitations of Phytoestrogens

Current evidence suggests that phytoestrogens may represent a promising complementary strategy for managing vasomotor and metabolic symptoms during the menopausal transition, particularly due to their estrogenic, antioxidant, and anti-inflammatory properties. Their biological activity appears to be strongly influenced by intestinal microbiota mediated metabolism, highlighting the relevance of compounds such as equol and other bioactive metabolites in modulating endocrine and metabolic homeostasis. Although several clinical studies report modest improvements in menopausal symptoms, lipid metabolism, and vascular function, findings remain heterogeneous because of differences in study design, dosage, duration, and individual microbial variability. Overall, these observations support the growing relevance of integrative nutritional approaches in climacteric care, while emphasizing the need for larger standardized and longitudinal studies to better establish long-term efficacy and clinical applicability.

Nevertheless, the study reported by [89], which investigated the combined effects of Black Cohosh, Soy Isoflavones, and SDG Lignans on menopausal symptoms, demonstrated more promising results. In this study, 96 women aged between 45 and 60 years were randomly assigned to receive either the active treatment or a placebo for a period of 90 days, with the aim of evaluating menopause-related symptoms. The results showed that, by the end of the intervention period, the final cohort consisted of 90 participants who completed the treatment. The combination of Black Cohosh, Soy Isoflavones, and SDG Lignans significantly reduced menopausal symptoms and exhibited a favorable safety profile. However, further systematic studies focusing on long-term follow-up are needed to better assess the effects of specific phytoestrogens on vasomotor symptoms. Although the isolation and procurement of specific phytoestrogens are complex and costly, it is feasible to evaluate targeted dietary interventions in specific regions. Such approaches could assess not only isoflavones but also other bioactive compounds, including amino acids such as L-isoleucine, which can be found in foods like broccoli [90] and may contribute to maintaining health during this stage by supporting cellular metabolism and homeostasis [91]. In the next section, we explain the different types of diets and how the microbiota interacts with the endocrine system to modulate hormonal activity, particularly estrogen metabolism.

#### 3.3.3. Gut Microbiota and Human Health

The gut microbiota comprises trillions of microorganisms that play a fundamental role in the body’s homeostasis, including metabolic, immunological, and endocrine functions. In recent years, it has been shown that the composition and diversity of the gut microbiota significantly influence the development of chronic diseases [92], including obesity, type 2 diabetes, cardiovascular diseases, and cancer [93,94,95,96]. One of the key mechanisms by which microbiota exerts its effects is through the production of metabolites, such as short-chain fatty acids (SCFAs), which regulate inflammation, intestinal permeability, and energy metabolism [97]. Additionally, the microbiota interacts with the endocrine system, modulating hormonal activity, including estrogen metabolism.

The term estrobolome refers to the collection of intestinal bacterial genes capable of metabolizing estrogens. These bacteria produce enzymes such as *β*-glucuronidase, which participate in the deconjugation of estrogens in the intestine, allowing their reabsorption through enterohepatic circulation [98,99]. Alterations in the composition of the estrobolome can lead to imbalances in circulating estrogen levels, which have been associated with various pathologies, including breast cancer, metabolic syndrome, and endocrine diseases [100,101,102,103]. In this context, diet emerges as a key modulating factor of the gut microbiota and, therefore, of estrogen metabolism.

Diet is one of the main determinants of gut microbiota composition. Diets rich in dietary fiber, polyphenols, and plant-based foods promote greater microbial diversity and support the growth of beneficial bacteria, whereas diets high in saturated fats and refined sugars may contribute to dysbiosis and altered host metabolic regulation [104]. Healthy dietary patterns such as the Mediterranean diet, the DASH diet, and plant-based diets have been associated with beneficial effects on gut microbiota composition, contributing to improved metabolic and inflammatory regulation [101,105].

#### 3.3.4. Mediterranean Diet, Microbiota, and Estrogen Metabolism

The Mediterranean diet is characterized by a high intake of fruits, vegetables, legumes, whole grains, olive oil, and fish, along with a low intake of red meat and ultra-processed foods. It is consistently associated with increased microbial diversity and a greater abundance of beneficial bacteria such as *Faecalibacterium prausnitzii*, *Bifidobacterium*, and *Eubacterium rectale*, as well as higher production of short-chain fatty acids (SCFAs), especially butyrate [106,107]. These changes are linked to reduced systemic inflammation and improved metabolic profiles [108,109]. Key components such as fiber, polyphenols, and unsaturated fatty acids promote microbial diversity and SCFA production, both of which are associated with metabolic benefits. Additionally, emerging evidence suggests that the Mediterranean dietary pattern may indirectly influence estrogen metabolism through modulation of the gut microbiota and the estrobolome, with potential implications for endocrine and metabolic homeostasis [110].

#### 3.3.5. DASH Diet and Gut Microbiota

The DASH diet (Dietary Approaches to Stop Hypertension) was developed to control high blood pressure and is based on a high intake of fruits, vegetables, low-fat dairy products, and whole grains, along with reduced sodium consumption. This diet has proven effective in lowering blood pressure and improving cardiovascular risk factors [111]. Although evidence regarding its direct impact on estrogen metabolism is limited, recent studies indicate that the DASH diet may also favorably modify gut microbiota composition by increasing beneficial bacteria associated with anti-inflammatory effects [112]. Therefore, its effect on the estrobolome may be indirect, mediated by changes in the microbiota.

While it shows positive effects on the gut microbiota, evidence is more limited compared with the Mediterranean diet. An increase in fiber-fermenting bacteria such as Prevotella has been observed, along with improved production of anti-inflammatory metabolites. However, evidence regarding its specific impact on estrogen metabolism remains limited [104].

#### 3.3.6. Plant-Based Diets, Microbiota, and Estrogens

Plant-based diets, including vegetarian and vegan patterns, are characterized by a high intake of fiber, antioxidants, and bioactive compounds. These diets are associated with greater microbial diversity and increased production of beneficial metabolites such as SCFAs [108]. The high-fiber intake in these diets may play an important role in regulating estrogen metabolism by reducing enterohepatic recirculation through increased fecal excretion [87]. Additionally, vegetarian diets have been shown to decrease circulating estrogen levels, which may reduce the risk of hormone-dependent diseases [113].

Plant-based diets are associated with marked changes in gut microbiota, characterized by a significant increase in microbial diversity and SCFA production [114]. This effect is mainly attributed to their high content of dietary fiber and bioactive compounds. Regarding estrogen metabolism, the literature indicates that the gut microbiota, through the estrobolome, regulates enterohepatic recirculation of estrogens via enzymes such as *β*-glucuronidase; in this context, plant-based diets have been shown to reduce circulating estrogen levels by increasing fecal excretion, potentially lowering the risk of hormone dependent diseases [97,99].

#### 3.3.7. Key Insights on Phytoestrogens and Nutritional Approaches

The evidence suggests that phytoestrogens and dietary patterns exert their effects not only through direct estrogen receptor interactions, but also via modulation of the gut microbiota and the estrobolome, influencing systemic estrogen availability and metabolic regulation. While clinical benefits on vasomotor symptoms and cardiometabolic markers appear modest and heterogeneous, integrative nutritional strategies (particularly those promoting microbial diversity) emerge as promising modulators of endocrine and metabolic homeostasis during the climacteric transition. This perspective supports a shift from isolated supplementation toward diet-based, microbiota informed interventions, although further longitudinal and standardized studies are required to clarify long-term efficacy and clinical applicability.

### 3.4. Physical Activity and Exercise Prescription

#### 3.4.1. Pathophysiological Rationale for Exercise in the Climacteric Transition

The climacteric period, encompassing perimenopause and postmenopause, is characterized by declining ovarian function, reduced estrogen levels, and consequent physiological changes that increase cardiometabolic risk. Women typically experience redistribution of adipose tissue toward visceral depots, sarcopenia, accelerated bone resorption, dyslipidemia, chronic low-grade inflammation, and heightened oxidative stress. These alterations contribute to a higher prevalence of obesity, osteoporosis, cardiovascular disease, and metabolic syndrome. Hormone therapy, while effective for vasomotor symptoms, is not universally suitable due to contraindications and patient preferences. Physical activity emerges as a safe, accessible, and evidence-based non-pharmacological strategy to mitigate many of these changes. This section reviews recent randomized controlled trials (RCT) examining aerobic training (AT), resistance training (RT), high-intensity interval training (HIIT), and combined modalities in climacteric women, with particular attention to effects on body composition, bone mass, lipid profile, oxidative stress, and inflammation.

#### 3.4.2. Aerobic Training

Aerobic training, involving sustained moderate-intensity activities such as walking, cycling, or dancing, remains the most studied modality in climacteric women. Moderate-intensity walking (60–70% of maximum heart rate (HRmax), 3–5 sessions/week) over 12–24 weeks consistently reduces adiposity and improves cardiovascular markers. A 2023 RCT demonstrated that 12 weeks of brisk walking significantly lowered body mass index (BMI) and waist circumference in postmenopausal women with obesity, alongside reductions in vascular inflammatory factors: interleukin-6 (IL-6), tumor necrosis factor-alpha (TNF-α), and C-reactive protein (CRP) [115]. Similarly, jazz dancing protocols (3 sessions/week, 60 min) over 16 weeks improved body composition (reduced fat mass, increased lean mass), muscle strength, and sleep quality, with sustained benefits at 6-month follow-up [116]. Recreational team handball, which incorporates aerobic elements with intermittent high-intensity bursts, enhanced aerobic performance, reduced fat mass, and improved cardiometabolic fitness markers after 16 weeks [117]. Jazz dance combined with concurrent training has also shown promise in alleviating broader menopausal symptoms while positively impacting body composition and inflammatory profiles [118].

Pilates-based aerobic training, despite improving maximum oxygen consumption (VO_2max_), has shown inconsistent effects on lipid parameters in dyslipidemic elderly postmenopausal women, suggesting that volume and intensity thresholds are critical [119]. Overall, aerobic training volumes of 150–300 min/week at moderate intensity appear sufficient to induce 1–3 kg fat mass loss and preserve lean mass, with greater benefits when energy expenditure exceeds 1200 kcal/week [115,116,117].

#### 3.4.3. Resistance Training

Resistance training directly counters sarcopenia and bone loss by stimulating muscle protein synthesis and osteogenesis through mechanical loading. Progressive RT (8–12 repetitions, 70–80% of one-repetition maximum (1RM), 2–3 sessions/week) using free weights, machines, or elastic bands has yielded robust outcomes. Twelve weeks of resistance band training in postmenopausal women with stage 1 hypertension reduced blood pressure, improved body composition (decreased fat mass, increased muscle mass), and attenuated age-related hormonal decline (insulin-like growth factor-1 (IGF-1) preservation) [120]. Similar protocols prevented metabolic syndrome progression in obese postmenopausal women [121] and enhanced physical function when combined with fish oil supplementation [122]. Short-term RT programs with varied intensities have effectively prevented sarcopenia progression, preserving lean mass and function [123].

Elastic band and whole-body vibration protocols have preserved or increased regional bone mineral density (BMD), particularly at the hip and spine [124,125]. High-load versus low-load RT comparably reduced inflammatory biomarkers (CRP, IL-6) while increasing muscle strength and mass [126]. Neuromuscular training variants, incorporating balance and power elements, improved regional BMD in early postmenopausal women [127]. These findings underscore RT as the cornerstone intervention for preserving muscle and bone, with elastic bands offering a practical, home-based alternative for women with limited gym access. Additionally, RT influences acute glucose tolerance, with effects modulated by aerobic capacity and baseline body composition [128].

#### 3.4.4. High-Interval Training and Combined Training

High-intensity interval training and combined training (CT) protocols, integrating aerobic and resistance elements, offer time-efficient alternatives with superior cardiometabolic outcomes in some populations. Sprint interval training (SIT; 6–8 × 30 s all-out efforts) reduced total and abdominal fat mass in postmenopausal women after 12 weeks [129]. Comparative trials showed CT (HIIT + RT) superior to HIIT alone for improving physical function markers (body fat percentage—0.5% reduction, chair stand—3 s augmentation, increased leg lean mass (0.3 kg), increased muscle strength (29%) and fast walking speed—5% augment) in obese postmenopausal women [130]. Concurrent training over 12–16 weeks favorably modified gut microbiota augmenting *β*-diversity and body composition in overweight/obese postmenopausal individuals [131].

Combined approaches incorporating impact loading (jumping, hopping) with RT have proven particularly effective for bone health. Twelve-month interventions of high-impact exercise (intermittent or continuous) increased hip and spine BMD in early postmenopausal women [132]. Bone-targeted RT with or without antiresorptive medication reduced fracture risk (up to 5% and 9.1%, respectively) indices in women with low bone mass [133,134,135]. These data suggest that protocols alternating high-impact aerobic intervals with resistance sets maximize osteogenic and anabolic stimuli while remaining feasible for most climacteric women.

#### 3.4.5. Effects of Exercise on Body Composition

Body composition deteriorates markedly during the climacteric transition, with average gains of 0.5–1 kg fat/year and losses of 0.3–0.5% muscle mass/year. Exercise consistently attenuates these changes. Trends across recent RCTs indicate that AT reduces total fat mass by 1–4%, visceral fat by 5–10%, and BMI by 1–2 kg/m^2^ [115,116,131,136]. Resistance training preferentially preserves or increases lean mass (0.5–2 kg) while reducing fat mass comparably or superiorly to AT in some trials [120,121,126,137]. High-intensity interval training and CT protocols elicit the most pronounced reductions in abdominal and visceral adiposity, likely via heightened post-exercise energy expenditure and catecholamine-driven lipolysis [129,130]. Intermittent fasting combined with exercise further optimizes body composition shifts, enhancing fat loss and physical performance [138].

Notably, exercise-induced changes in body composition occur independently of significant weight loss, reflecting improved partitioning toward lean tissue. Adherence-enhancing strategies, such as supervised group sessions or progressive overload, amplify outcomes up to 58.9% of average adherence to exercises [139].

#### 3.4.6. Effects of Exercise on Bone Mass

Postmenopausal bone loss averages 1–2% annually at the spine and hip, accelerating fracture risk. Mechanical loading via weight-bearing and resistance exercise stimulates osteoblast activity and suppresses osteoclastogenesis. High-impact protocols (multidirectional hopping, jumping) increase hip and spine BMD by 1–3% over 12 months through intermittent mechanical loading that stimulates bone adaptation and remodeling, countering postmenopausal bone loss by activating osteoblasts and preserving or modestly enhancing density in weight-bearing sites [132,134,135]. Resistance training with progressive loading (70–85% 1RM) similarly preserves or increases BMD, particularly when combined with impact [133,140]. Whole-body vibration and elastic band protocols yield modest but significant gains in postmenopausal osteoporosis by applying mechanical oscillations that enhance muscle strength and stimulate bone remodeling, resulting in improved lumbar and femoral bone mineral density through increased osteogenic activity. Elastic band resistance exercises, often combined with traditional movements like Yi Jin Jing, provide progressive loading that delays bone mineral density decline across the body by promoting osteoblast function and overall skeletal health in postmenopausal women [124,125].

Aerobic exercise alone (walking, dancing) generally maintains rather than increases BMD, though it reduces fall risk (by 14.3% overall, reductions of 38% in fractures and 41% in severe injury falls) through improved balance and strength [136,141]. Combined impact + RT interventions appear most efficacious, with effects comparable to or additive with bisphosphonates in some trials [133,134].

#### 3.4.7. Effects of Exercise on Lipid Profile and Cardiometabolic Markers

Dyslipidemia affects up to 70% of postmenopausal women, characterized by elevated low-density lipoprotein cholesterol (LDL-C), triglycerides, and reduced high-density lipoprotein cholesterol (HDL-C). Exercise improves lipid parameters primarily through enhanced lipoprotein lipase activity and reverse cholesterol transport. Moderate-intensity AT and recreational sports (handball, jazz dance) consistently lower total cholesterol and triglycerides while raising HDL-C [116,117,119]. Resistance band exercise training in obese postmenopausal women reduced LDL-C by 10.8 mg/dL and improved insulin sensitivity, including reductions of 0.6 units in HOMA-IR, 1.3 μU/mL in fasting insulin, and 4.5 mg/dL in fasting glucose [121]. In hypertensive postmenopausal women, resistance-band training additionally improved blood pressure, body composition, and age-related hormonal profiles [120]. Combined training and HIIT show superior triglyceride reductions and HDL-C elevations, likely due to greater caloric expenditure and muscle mass gains [130,131]. Estrogen levels modulate these metabolic responses to RT, with greater benefits in early postmenopause [142]. Supplemented trials (fish oil, royal jelly) suggest additive benefits on lipid peroxidation markers, but exercise alone drives most improvements [122,143].

#### 3.4.8. Role of Exercise in Reducing Oxidative Stress and Inflammation

Chronic low-grade inflammation and oxidative stress accelerate cardiometabolic and musculoskeletal decline during climacteric transition. Elevated CRP, IL-6, TNF-α, and reduced antioxidant capacity (paraoxonase-1, superoxide dismutase) are common. Walking-based AT significantly reduces vascular inflammatory factors and CRP [115]. Resistance training at varying loads decreases systemic inflammation markers. RT alone induced a significant decrease in the TNF-α, with log10-transformed changes of −0.86 (95% CI: −1.31 to −0.43) in the low-load group (pre: 0.52 ± 0.24, post: −0.34 ± 1.1) and −0.34 (95% CI: −0.76 to 0.07) in the high-load group (pre: 0.49 ± 0.36, post: 0.14 ± 0.41), and induced a significant decrease in the pro-inflammatory marker MCP-1 f (rom 253 ± 119 to 206 ± 106 pg/mL), alongside a decrease in adiponectin from 584 ± 572 to 245 ± 356 pg/mL. [124,144]. Combined aerobic-resistance protocols enhance paraoxonase-1 activity (1.1%) and liver function (reductions in both ALT and AST, showing significant time per group interactions) in women with metabolic dysfunction [143].

Mechanistically, exercise upregulates nuclear factor erythroid 2-related factor 2 (Nrf2) signaling, boosts endogenous antioxidants (glutathione peroxidase, catalase), and reduces nicotinamide adenine dinucleotide phosphate (NADPH) oxidase activity. Regular training also lowers visceral fat-derived pro-inflammatory cytokines. Although fewer trials directly measure oxidative stress, consistent reductions in 8-hydroxy-2′-deoxyguanosine (8-OHdG), malondialdehyde, and advanced oxidation protein products accompany body composition improvements [145].

#### 3.4.9. Key Insights on Physical Activity and Exercise Prescription

Table 4 summarizes the effects of different exercise interventions in postmenopausal women, showing that aerobic, resistance, and combined training improve body composition, cardiometabolic health, and physical function. Additionally, high-intensity and bone-targeted exercises contribute to fat reduction and increased bone mineral density, highlighting the role of exercise as a key non-pharmacological strategy during postmenopause.

The body of evidence reviewed demonstrates a clear and consistent positive effect of exercise interventions on cardiometabolic and musculoskeletal health in postmenopausal women. Across diverse modalities (including aerobic, resistance, high-intensity, and combined training) most studies report improvements in body composition, physical performance, metabolic regulation, and, in several cases, bone health outcomes. These benefits are observed despite considerable variation in intervention duration, frequency, and exercise protocols.

Importantly, the consistency of favorable outcomes across heterogeneous study designs reinforces the robustness and clinical relevance of exercise as a key non-pharmacological strategy during the menopausal transition. While methodological differences limit the identification of a single optimal exercise prescription, they do not diminish the overall strength of the evidence supporting regular physical activity.

Therefore, exercise should be considered an important component in the prevention and management of cardiometabolic risk in postmenopausal women. Future research should aim to refine and standardize intervention protocols to further optimize individualized recommendations and maximize long-term health benefits.

## 4. Discussion

### 4.1. Integrated Perspective on Sarcopenia and Sarcopenic Obesity During the Climacteric Transition

Taken together, the available evidence indicates that the climacteric transition should be understood not as an isolated hormonal event, but as a multifactorial cardiometabolic process characterized by dynamic interactions among adiposity, endocrine changes, metabolic regulation, and alterations in body composition. In this context, the coexistence of increased visceral adiposity and progressive declines in skeletal muscle mass has led to growing recognition of sarcopenic obesity as a clinically relevant phenotype during the menopausal transition, with important implications for metabolic and functional health [25,26,36,44].

One of the most consistent findings across studies is the central role of visceral adiposity (VAT) as a key contributor to cardiometabolic risk. Evidence derived from large observational cohorts, imaging-based investigations, and multicenter DXA analyses consistently indicates that VAT, rather than total body weight, exhibits the strongest associations with metabolic syndrome components and cardiovascular risk factors [22,30,41]. Notably, multicenter DXA-based analyses have shown that up to 22% of women with a normal body mass index present elevated visceral adiposity, while more than one-third exhibit an android fat distribution pattern, underscoring the limitations of BMI as a surrogate marker of cardiometabolic risk [41].

Across the menopausal transition, evidence from observational studies, multicenter DXA analyses, and body composition investigations consistently indicates that changes in body composition are characterized not only by weight gain but also by a preferential redistribution of fat toward central and visceral depots [22,41,44]. Importantly, these alterations may occur despite relatively stable BMI values, highlighting the limitations of BMI as a marker of metabolically relevant adiposity and cardiometabolic risk [22,41,44]. Furthermore, findings across different study populations suggest that fat distribution may be more relevant than absolute fat mass, with upper-body adiposity consistently associated with greater cardiometabolic risk, whereas lower-body fat may exert protective effects [22].

Evidence from a large population-based NHANES analysis including 4061 postmenopausal women supports the relevance of long-term weight trajectories as indicators of cardiometabolic risk [39]. Unlike cross-sectional anthropometric measures, longitudinal BMI patterns capture cumulative exposure to adiposity and may therefore provide additional insight into metabolic vulnerability during the menopausal transition. These observations reinforce the importance of adopting a life-course perspective when evaluating cardiometabolic health in midlife women.

Beyond overall adiposity, evidence from cross-sectional studies, multicenter DXA analyses, and obesity-focused investigations involving more than 1500 women collectively suggests that cardiometabolic risk is more accurately reflected by composite metabolic indices than by conventional anthropometric measures [32,41,42]. Indices such as the visceral adiposity index (VAI), lipid accumulation product (LAP), and body roundness index (BRI) integrate information on fat distribution and metabolic parameters, providing a more comprehensive characterization of cardiometabolic dysfunction than BMI alone. This distinction is particularly relevant in postmenopausal women, in whom BMI may fail to identify clinically meaningful alterations in fat distribution and metabolic risk, potentially leading to an underestimation of cardiometabolic vulnerability [32,41,42].

The role of inflammation and adipokines appears to be more nuanced than traditionally assumed. Although evidence from a meta-analysis including 7207 pre- and postmenopausal women indicated that increases in adiposity are not consistently accompanied by elevations in systemic inflammatory markers, with IL-6, CRP, and TNF-α showing variable or non-significant associations across studies [36], these findings suggest that the relationship between fat accumulation and systemic inflammation during the menopausal transition may be more complex than previously recognized. Nevertheless, evidence from an observational study including 240 postmenopausal women demonstrated significant associations between TNF-α, IL-6, insulin resistance, and metabolic dysfunction [38]. These findings indicate that inflammatory pathways may contribute to cardiometabolic risk in specific populations, despite the heterogeneity observed across studies.

In contrast, adipokines appear to exhibit more consistent associations with metabolic dysfunction across studies. In a cross-sectional study including 168 peri- and postmenopausal women, adiponectin was inversely associated with insulin resistance, triglyceride levels, and indices of visceral adiposity, while showing positive associations with HDL-cholesterol [37]. These findings support the relevance of adipokine regulation as a component of the metabolic alterations accompanying the menopausal transition and suggest a potentially important role for adiponectin in maintaining metabolic homeostasis.

Hormonal changes during menopause interact closely with body composition and metabolic regulation. Evidence from a cross-sectional study including 133 postmenopausal women suggests that the associations between endogenous sex hormones and cardiovascular risk markers may vary according to adiposity status, indicating that body composition can influence the cardiometabolic effects of the hormonal milieu [29]. These findings highlight the complex interplay between hormonal changes and adiposity during the menopausal transition and may partly explain the heterogeneity in cardiometabolic risk observed among midlife women.

Importantly, not all metabolic changes observed during the climacteric transition can be attributed solely to hormonal decline. Evidence from a 24-month prospective controlled cohort study including 104 postmenopausal women indicated that estrogen suppression through aromatase inhibitor therapy was not independently associated with adverse changes in adiposity or cardiometabolic risk [31]. These findings suggest that aging, body fat redistribution, and lifestyle-related factors may contribute alongside hormonal changes to cardiometabolic dysfunction. Taken together, evidence derived from large cohort studies, multicenter DXA analyses, meta-analyses, and population-based investigations supports an integrative model in which hormonal, metabolic, and body composition factors interact to influence cardiometabolic risk during the menopausal transition [22,36,39,41].

Another key aspect emerging from the literature is the progressive nature of cardiometabolic risk following menopause. Evidence from a cross-sectional study including 705 postmenopausal women demonstrated that longer duration since menopause was associated with a higher prevalence of metabolic syndrome and a less favorable metabolic profile [34]. These findings suggest that cardiometabolic burden may accumulate over time rather than occurring abruptly during the menopausal transition.

These temporal patterns underscore a potential window for early intervention, as metabolic alterations appear to intensify with advancing postmenopausal duration. Taken together, the available evidence supports a conceptual framework in which cardiometabolic vulnerability during the menopausal transition is influenced not only by traditional risk factors, but also by fat distribution, composite metabolic indices, menopausal timing and duration, and long-term weight trajectories [22,32,34,39,41,42]. Integrating these dimensions may provide a more comprehensive understanding of cardiometabolic risk in midlife women.

Lifestyle interventions, including dietary modification and structured physical activity, represent important strategies for mitigating cardiometabolic vulnerability during the menopausal transition. The available evidence suggests that interventions targeting body composition and metabolic health may contribute to improvements in adiposity, insulin sensitivity, and overall cardiometabolic profiles in postmenopausal women [35,45,47,48,50]. In addition, selected evidence indicates that hormonal therapy may provide complementary cardiometabolic benefits in appropriately selected women, particularly when initiated during early postmenopause [35].

Overall, the available evidence supports a broader understanding of cardiometabolic health during the menopausal transition, moving beyond a static, hormone-centered model toward a dynamic, integrative, and life-course–based framework. This perspective highlights the interaction among hormonal changes, adiposity, metabolic regulation, and lifestyle factors, and provides a conceptual basis for understanding the heterogeneity of cardiometabolic risk observed among midlife women [22,35,36,39,41].

### 4.2. Integrative Perspective on Heart Rate Variability Changes During Menopause

Heart rate variability remains a robust, non-invasive indicator of cardiovascular health as concluded in the review of Sundas et al. [52]. Nevertheless, the authors also underscore the lack of standardized protocols, which makes it difficult to compare quantitative results, and indicate the presence of contradictions on the results among studies. When HRV is presented as an indicator on the cardiovascular health of the effects of hormone changes due to the menopausal transition in women, one of the main sources of debate lies in distinguishing the relative contributions of aging versus menopausal hormonal changes. For example, Solanky et al. [58] studied PRE, PERI and PM in a narrow age range (40–55 yrs.) and found no HRV differences among age-matching groups; however, the study lacked data of hormone levels. Ramesh et al. [57] conducted a study that included estradiol levels and concluded that age rather than estradiol was the main factor driving for the HRV decline. De Jager [54] presented an intermediate position in a review, where the authors found that HRV varies with hormone fluctuations and declines with both age and menopause. Similarly, von Holzen et al. [55] concluded in another review that HRV indeed decreases with menopause, but hormone therapy partially restores it. However, age was not discussed at all in this review. Carvalho et al. [56] used a mixed approach, considering early (<6 yrs) and late (>6 yrs) PM women. They observed the decrease in HRV with time of menopause although the effect of group age was mentioned but not discussed. One way to disentangle hormonal from age-related effect is to include men groups as control as done by Voss et al. [82], who found the disappearance of gender HRV differences that were present in young groups but diminished with age. Most studies document, or take for granted, a postmenopausal decline in HRV and a shift toward sympathetic dominance. However, the natural correlation between age and menopause transition may induce confusion. Consequently, age-matched comparisons frequently attribute part or most of this reduction to chronological age rather than reproductive status alone.

Another potential indicator of menopause is the presence and severity of menopausal symptoms. Stokes et al. [69] reported higher HRV in the symptomatic group. However, Martinelli et al. [70] concluded the HRV decreases with symptom severity. Similarly, Sahu et al. [71] reported the usual decrease in HRV in PM women with an even greater reduction in the symptomatic group. These conflicting findings, particularly regarding VMS and in some studies related with the efficacy of hormone therapy, likely stem from methodological differences, small sample sizes, and variable control for confounders such as comorbidities and lifestyle.

The inclusion of other factors in menopause research in general complicates the analysis and interpretation of the results. In particular, the addition of age-related comorbid conditions such as hypertension [72] and diabetes [74,75] appear to amplify the negative impact of menopause on autonomic function, whereas protective lifestyle factors including regular exercise [59,60,61,62] and sexual activity [81] support higher HRV. In the case of exercise, some negative results could be explained by its limitations, as in the study of Jones et al. [68] that found no association between HRV and VMS regardless of physical activity, but the recordings were not made during symptoms appearance. Takkar et al. [53] did not find relation between HRV and 6 min walking test, maybe because of the short time duration of the test.

In summary, this review indicates that HRV declines across the menopausal transition, driven predominantly by chronological age but further modulated by hormonal withdrawal, VMS, and other conditions and comorbidities. Key takeaways are that lifestyle interventions emerge as possible consistent cardioprotective factors, whereas hormone therapy effects on HRV remain nuanced and regimen dependent. Clinically, routine HRV assessment in general is proposed as a practical tool for identification of autonomic imbalance and elevated cardiovascular risk in postmenopausal women, especially when other factors or health conditions are involved as hypertension, diabetes, or insomnia, among others. The above investigations are just some examples of the recent research tendency in this topic. Therefore, the potential cardioprotective factors cannot be considered specific clinical recommendations, as they have not been thoroughly reviewed, and, as most of the results reviewed are cross-sectional studies, they do not imply cause–effect relationships.

In this review about HRV in menopause stages, the main limitation is that the publications included did not undergo rigorous quality evaluation. However, the publication search underwent a formal methodology in terms of time, keywords, and database used, as explained before. Most of the studies are transversal or observational with few longitudinal studies (those that include exercise, for example). In some studies, HRV is not the focus but one of many indices regarding the cardiovascular status of the subject and menopause is also, in some cases, just one of the factors involved. We also include some of the recent representative investigations of ongoing research in the field. These investigations address diverse common factors related to age and menopause and were included to explore evidence of menopause as an unquestionable factor of HRV decline.

Future research should adopt standardized HRV protocols and main indices, incorporate age- and gender-matched controls, and employ longitudinal designs to more clearly separate hormonal from aging contributions. Such efforts will strengthen evidence-based management of cardiovascular health during and beyond menopause.

### 4.3. Gut Microbiota, Estrobolome, and Cardiometabolic Health During the Climacteric Transition

Emerging evidence suggests that the gut microbiota may play an important role in the complex interactions among estrogen metabolism, dietary factors, and cardiometabolic health during the climacteric transition. Although phytoestrogens have traditionally been investigated for their potential effects on vasomotor symptoms (VMS) and other manifestations of estrogen deficiency [84,91], increasing attention has been directed toward their possible influence on metabolic and inflammatory pathways that contribute to cardiometabolic risk in midlife women.

The gut microbiota functions as a metabolically active ecosystem capable of influencing endocrine, immune, and metabolic homeostasis. In this context, dietary patterns rich in fiber, polyphenols, fruits, vegetables, legumes, and whole grains appear to promote greater microbial diversity and stimulate the production of short-chain fatty acids (SCFAs), particularly butyrate, which have been associated with anti-inflammatory and metabolic benefits. Because phytoestrogens require microbial biotransformation to generate biologically active metabolites, the composition and functionality of the gut microbiota may partially determine individual variability in the physiological response to these compounds [91]. This interaction may help explain the heterogeneous findings reported across studies evaluating the effectiveness of phytoestrogens in menopausal women.

The potential cardiometabolic relevance of these mechanisms is supported by evidence linking dietary modulation of the microbiota with improvements in inflammatory and metabolic profiles. The NU-AGE trial conducted by Ghosh et al. [104] demonstrated that adherence to a Mediterranean diet for 12 months increased the abundance of microorganisms associated with lower frailty and reduced inflammatory biomarkers, including C-reactive protein and interleukin-17. Furthermore, increased SCFA production and lower concentrations of potentially harmful metabolites were observed. Although the primary outcomes of this study were not specifically cardiometabolic, these findings support the hypothesis that microbiota-mediated dietary effects may contribute to metabolic homeostasis and reduction in chronic low-grade inflammation, both of which are recognized contributors to cardiometabolic risk during the menopausal transition.

Similarly, Pieczyńska and Rzymski [102] reported that long-term adherence to a Mediterranean dietary pattern was associated with greater microbial diversity and a more favorable metabolic profile. These observations are consistent with the growing body of evidence suggesting that dietary interventions may influence cardiometabolic health not only through direct nutritional effects but also through modulation of the gut microbiome [93,99,100,104,108,111]. Nevertheless, considerable heterogeneity exists across studies regarding dietary exposures, microbiota characterization methods, participant characteristics, and clinical outcomes, limiting direct comparisons and precluding definitive conclusions regarding causality [93,100,102,104,108,111].

Particular interest has recently focused on the estrobolome, defined as the collection of intestinal microorganisms capable of metabolizing estrogens through *β*-glucuronidase activity [98]. Through the regulation of enterohepatic estrogen recirculation, the estrobolome may influence circulating estrogen concentrations and thereby affect multiple physiological processes relevant to cardiometabolic health. Bolgova, Shypilova, and Mavrych [87] suggest that microbiota-targeted interventions, including probiotics, may influence estrogen availability in postmenopausal women. However, the magnitude and clinical significance of these effects remain uncertain, and the underlying mechanisms require further investigation in well-designed longitudinal studies.

Although the concept of the estrobolome has generated considerable interest, current evidence remains largely observational and mechanistic. As highlighted by Larnder, Manges, and Murphy [98], alterations in microbial estrogen metabolism have been associated with several estrogen-related conditions, yet robust prospective studies evaluating clinically relevant cardiometabolic outcomes remain scarce. Consequently, it is premature to establish direct causal relationships between estrobolome modulation and cardiometabolic disease prevention.

Taken together, the available evidence supports the hypothesis that interactions among diet, gut microbiota, phytoestrogen metabolism, and estrogen homeostasis may contribute to cardiometabolic regulation during the climacteric transition. However, substantial heterogeneity exists regarding study populations, dietary exposures, microbiota characterization methods, and clinical outcomes. As emphasized by Perrone and D’Angelo [100], larger randomized controlled trials with longer follow-up periods are needed to clarify whether targeted modulation of the gut microbiota and the estrobolome can produce clinically meaningful improvements in cardiometabolic health among menopausal women.

### 4.4. Exercise-Mediated Mechanisms and Cardiometabolic Benefits During the Climacteric Transition

The reviewed RCTs demonstrate consistent cardiometabolic benefits of structured exercise in climacteric women, although magnitude and consistency vary by modality and menopausal stage. Moderate-intensity aerobic training (brisk walking or jazz dance, 12–16 weeks) significantly reduced BMI, waist circumference, fat mass, and inflammatory markers (IL-6, TNF-α, CRP) in obese postmenopausal women [115,116], whereas Pilates protocols produced inconsistent lipid improvements in dyslipidemic elderly participants, indicating that volume and intensity thresholds are critical [119]. Resistance training (progressive elastic-band or machine-based, 70–80% 1RM, 2–3 sessions/week) reliably decreased fat mass, preserved lean mass and IGF-1 levels, lowered blood pressure, and attenuated CRP, IL-6, and TNF-α across hypertensive and obese cohorts [120,126]; high- versus low-load protocols yielded comparable anti-inflammatory effects [126]. Combined training (HIIT + RT) outperformed HIIT alone, producing greater reductions in body fat percentage, increases in leg lean mass, muscle strength, and walking speed in obese postmenopausal women [130], while sprint interval training effectively lowered visceral and total fat mass [129].

Physiological mechanisms supporting these outcomes include enhanced endothelial function and reduced visceral adipose-derived pro-inflammatory cytokines with aerobic training; mechanical loading from resistance exercise stimulating muscle protein synthesis, osteoblast activity, and preservation of IGF-1 [120]; catecholamine-driven lipolysis and elevated post-exercise energy expenditure (EPOC) promoting greater fat oxidation with HIIT [129,130]; and gut microbiota modulation (increased *β*-diversity) improving metabolic efficiency with concurrent training [131]. Across modalities, exercise upregulates Nrf2 signaling, boosting endogenous antioxidants (glutathione peroxidase, catalase) while suppressing NADPH oxidase activity and oxidative stress [145], alongside enhanced lipoprotein lipase activity and reverse cholesterol transport that improve lipid profiles. Estrogen status further modulates responses, with greater metabolic adaptations to resistance training observed in early postmenopause [142]. Acute glucose tolerance improvements after resistance exercise are influenced by baseline aerobic capacity and body composition [128].

Limitations include substantial heterogeneity in intervention duration (8–52 weeks), intensity, populations (early vs. late postmenopause), and adherence, plus scarce long-term data on clinical events (CVD, fractures) and few direct head-to-head comparisons, precluding firm dose–response conclusions.

Clinically, these findings support multimodal exercise prescriptions (moderate aerobic activity [150–300 min/week] combined with progressive resistance training [2–3 sessions/week] and impact loading when feasible) as a safe, effective strategy to mitigate cardiometabolic risk during the climacteric, especially when hormone therapy is contraindicated. Supervised or group formats may improve adherence [139].

Future research should prioritize standardized long-term RCTs with direct modality comparisons, stratification by estrogen status and baseline metabolic profile, and integration with nutritional interventions to develop personalized, sustainable cardiometabolic protection in diverse climacteric populations.

### 4.5. Strengths and Limitations

This review has several strengths. It integrates evidence from four complementary domains (body composition, heart rate variability, phytoestrogens and estrobolome-related mechanisms, and physical exercise interventions) providing a multidimensional perspective on cardiometabolic health during the climacteric transition. In addition, the review incorporates evidence from multiple databases and emphasizes recent literature while retaining seminal studies that contribute important conceptual and mechanistic insights.

However, several limitations should be acknowledged. First, this study was conducted as a narrative review, and therefore a formal quality assessment of the included studies was not performed. Consequently, the findings should be interpreted in light of the methodological heterogeneity of the available literature. Second, the reviewed studies varied substantially in design, sample characteristics, definitions of menopausal stage, outcome measures, and methods used to assess body composition, autonomic function, dietary exposures, and exercise interventions. Furthermore, differences in geographic settings, ethnic backgrounds, and healthcare contexts may limit the generalizability of some findings across populations. Third, a considerable proportion of the available evidence derives from observational and cross-sectional studies, limiting causal inference. Finally, despite the structured literature search framework employed, the possibility of selection bias and publication bias cannot be completely excluded. Therefore, future research would benefit from standardized methodologies, longitudinal designs, and well-controlled intervention studies to further clarify the mechanisms underlying cardiometabolic vulnerability during the climacteric transition.

## 5. Conclusions

The evidence synthesized in this review indicates that cardiometabolic risk during the climacteric transition arises from the convergence of adverse changes in body composition, autonomic regulation, and modifiable lifestyle factors. Increased visceral adiposity combined with progressive loss of lean mass defines a metabolically unfavorable phenotype that is not adequately captured by conventional anthropometric measures such as body mass index, underscoring the need for body composition-based assessment incorporating fat distribution, muscle mass, and functional capacity.

Autonomic dysfunction, reflected by reduced heart rate variability, represents a clinically relevant yet underrecognized component of cardiovascular risk. Although largely influenced by aging, HRV is further modulated by hormonal changes, sleep disturbances, vasomotor symptoms, and comorbidities. Current evidence supports its value as a non-invasive marker of autonomic function and cardiovascular risk; however, additional longitudinal and interventional studies are needed before routine risk stratification or personalized clinical management based on HRV can be recommended in climacteric women.

Nutritional and microbiota-related mechanisms add a further integrate dimension. Diets rich in plant-based foods and bioactive compounds promote gut microbiota diversity and influence estrogen metabolism through the estrobolome, suggesting a mechanistic link between diet, endocrine regulation, and cardiometabolic health, although more standardized evidence is required.

Among non-pharmacological strategies, physical activity emerges as the most consistently effective intervention. Across modalities, exercise demonstrates reproducible benefits in body composition, metabolic regulation, bone health, and autonomic function, supporting its role as a central pillar in prevention and management.

Collectively, these findings support a shift from isolated risk factor management toward an integrated, multidimensional approach in which cardiometabolic risk is understood as the result of dynamic interactions among hormonal, metabolic, autonomic, and behavioral factors. Future research should prioritize longitudinal and interventional designs with standardized methodologies to disentangle the relative contributions of aging, hormonal decline, and lifestyle, ultimately improving risk stratification and long-term outcomes in women undergoing the climacteric transition.

## Figures and Tables

**Table 1:** Multisystem Physiological Changes during the Climacteric Transition.

Domain	Physiological Changes	Underlying Mechanisms	Health Outcomes and Clinical Implications
Endocrine transition [1,2,3,4]	Progressive decline in estrogen and progesterone; neuroendocrine instability	Ovarian senescence; altered hypothalamic thermoregulation; changes in serotonergic and noradrenergic signaling	Vasomotor symptoms, sleep disturbances, mood-related symptoms, and altered autonomic regulation
Body composition [5,6]	Increased visceral adiposity and central fat redistribution	Estrogen deficiency–related adipocyte dysfunction and skeletal muscle decline	Central obesity, early sarcopenic changes, and increased metabolic vulnerability
Lipid and glucose metabolism [5,6,7,8]	Dyslipidemia and insulin resistance	Altered lipid handling, reduced insulin sensitivity, and a pro-inflammatory state	Increased risk of type 2 diabetes and cardiovascular disease
Autonomic regulation [10,11,14]	Reduced heart rate variability and sympathetic predominance	Estrogen-related autonomic modulation and stress-related physiological responses	Hypertension and impaired cardiovascular adaptation
Sleep and circadian health [12,13]	Sleep fragmentation and circadian disruption	Neuroendocrine instability and altered sleep–wake regulation	Weight gain, impaired glucose regulation, inflammation, and reduced overall well-being.
Cardiovascular function [1,5,7,10]	Endothelial dysfunction and increased systolic blood pressure	Endocrine alterations interacting with metabolic and inflammatory pathways	Accelerated atherosclerosis and vascular dysfunction
Metabolic syndrome (MetS) [8,12,15]	Increased prevalence after menopause (30–45%)	Clustering of metabolic abnormalities	Increased cardiovascular morbidity and mortality risk
Neuroautonomic and gastrointestinal responses [14,15]	Altered gastric motility and digestive function	Activation of the hypothalamic–pituitary–adrenal (HPA) axis and altered autonomic regulation	Gastrointestinal symptoms and associations with metabolic dysregulation
Integrated cardiometabolic profile [1,5,10,15,16]	Multisystem physiological alterations	Interaction among endocrine, metabolic, autonomic, and lifestyle-related systems	Reduced physiological adaptability and increased susceptibility to chronic disease burden

Abbreviations: MetS, metabolic syndrome; HPA, hypothalamic–pituitary–adrenal.

**Table 2:** Characteristics of included studies on body composition and cardiometabolic risk during the climacteric transition.

Author [Ref.]	Year	Study Design	Sample Size	Population	Main Outcome
Numao et al. [21]	2020	Cross-sectional	163	PRE/PM	VAT is associated with increased triglycerides, reduced HDL-C, and elevated glucose levels independently of menopausal status
Chen et al. [22]	2022	Cohort	155,925	PM	Upper-body adiposity is associated with increased cardiometabolic risk, whereas lower-body fat distribution appears to be protective
Gulbahar et al. [23]	2022	Cross-sectional (with 10-year follow-up)	50	PM	VAI is positively associated with metabolic syndrome and insulin resistance, but not with 10-year CVD incidence
Harraqui et al. [24]	2022	Cross-sectional	373	PERI/PM	Metabolic syndrome is strongly associated with abdominal obesity, particularly increased waist circumference, along with adverse metabolic parameters
Kodoth et al. [25]	2022	Narrative review	NA	Transition	Menopausal transition is associated with increased fat mass, reduced lean mass, and visceral fat redistribution, contributing to elevated cardiovascular risk
Marlatt et al. [26]	2022	Narrative review	NA	Transition	Central adiposity is a stronger predictor of cardiometabolic risk than BMI, with normal-weight central obesity conferring increased risk
Matta et al. [27]	2022	Observational case–control study	160	PRE/PM	Elevated hepcidin is associated with insulin resistance and abdominal adiposity
Miranda et al. [28]	2022	Cross-sectional	75	PM (MetS)	Handgrip strength predicts fat-free mass and is inversely associated with blood pressure in PM women with metabolic syndrome
Ottarsdottir et al. [29]	2025	Cross-sectional	133	PM	Endogenous sex hormones are associated with increased cardiovascular risk, highlighting hormonal modulation of cardiometabolic risk
Stamm et al. [30]	2022	Cross-sectional study (within a cohort)	803	PM (non-obese)	Visceral adipose tissue measured by DXA is the strongest predictor of cardiometabolic risk
Cheung et al. [31]	2023	Prospective cohort study (controlled, 24 months)	104	PM (52 AI/52 control)	Aromatase inhibitor therapy is not associated with adverse changes in adiposity or cardiometabolic risk, with parallel increases in visceral fat observed in both groups over time
El Shikieri et al. [32]	2023	Cross-sectional	224	PM	VAI and LAP are associated with CVD risk, with VAI showing superior predictive performance
Ospino et al. [33]	2023	Cross-sectional	205	PM	Increased adiposity, especially central fat accumulation, is linked to an adverse cardiometabolic profile marked by elevated triglycerides, reduced HDL-C, and a higher TG/HDL ratio
Erdogan et al. [34]	2024	Cross-sectional	705	PM	Menopause duration > 5 years is associated with a significantly higher risk of metabolic syndrome (OR 6.44; 95% CI 3.34–12.45)
Lambrinoudaki et al. [35]	2024	Narrative review	NA	PERI/PM	Menopause represents a cardiometabolic transition characterized by increased central adiposity, insulin resistance, and a pro-atherogenic profile
Pernoud et al. [36]	2024	Meta-analysis	7207	PRE/PM	PM status is associated with greater adiposity and higher adipokine levels, while inflammatory markers (IL-6, CRP, TNF-α) show inconsistent or non-significant changes
Cybulska et al. [37]	2025	Cross-sectional	168	PERI/PM	Adiponectin inversely associated with visceral adiposity indices and cardiometabolic risk markers, and positively with HDL
Khalaf et al. [38]	2025	Cross-sectional	240	PM	TNF-α and IL-6 positively associated with insulin resistance (HOMA-IR) and metabolic dysregulation
Liu et al. [39]	2026	Cross-sectional (NHANES)	4061	PM	Increasing and increasing–decreasing BMI trajectories were associated with higher metabolic syndrome risk (OR 5.09, 95% CI 3.19–8.13; OR 5.89, 95% CI 3.73–9.31)
Orhan et al. [40]	2025	Cross-sectional	150	PRE (74), PM (77)	PM status was associated with a less favorable cardiometabolic profile, including increased adiposity, blood pressure, and fasting glucose levels
Park et al. [41]	2025	Multicenter cross-sectional	914	PERI/PM	VAT and A/G ratio increased with age; 22% of women with normal BMI exhibited excess visceral adiposity
de Luis et al. [42]	2025	Cross-sectional	468	PM (obesity)	BRI showed significant predictive value for metabolic syndrome in postmenopausal women with obesity (AUC 0.75; OR 2.65; 95% CI 1.99–3.53)
Beydoun et al. [43]	2026	Prospective cohort study (median follow-up: 18 years)	91,392	PM	Anthropometric indices showed modest predictive value for diabetes risk (AUC < 0.60; c-statistics 0.58–0.59), with no clear superiority of novel over traditional measures
Szeliga et al. [44]	2026	Cross-sectional	325	PRE/PERI/PM	Menopausal transition is characterized by decreased lean mass and increased visceral adiposity, indicating a shift toward central fat accumulation
Félix-Soriano et al. [45]	2021	RCT	124	PM (71 completed)	Reduced body weight and fat mass; RT improved lean mass and glucose tolerance, DHA reduced triglycerides and blood pressure; no synergistic effects
Magalhães et al. [46]	2023	Cross-sectional	31	PM	MUH phenotype linked to dyslipidemia and visceral adiposity; age as a predictor despite resistance training
Cota e Souza et al. [47]	2024	RCT	102	Climacteric	Yoga reduced MetS prevalence by up to 46% and improved glucose, HDL-cholesterol, waist circumference, and blood pressure
Jóźwiak et al. [48]	2024	Quasi-experimental trial (12 weeks)	62	PM	Time-restricted eating combined with exercise improved adiposity and insulin resistance markers more than exercise alone
Ilich et al. [49]	2022	RCT	97 (30/37/30)	PM	Calcium & vitamin D supplementation or dairy intake enhances improvements in blood pressure, lipid profile, and adipokines during weight loss
Bajerska et al. [50]	2025	Cross-sectional	312	PM	Greater adherence to the Mediterranean diet was associated with lower odds of central obesity (OR 0.669; 95% CI 0.518–0.866) and hypertension (OR 0.817; 95% CI 0.689–0.969)
Veronese et al. [51]	2024	Narrative review	NA	Climacteric	MedDiet diet is associated with reduced inflammation and lower osteoarthritis risk

Abbreviations: PRE: premenopausal; PERI: perimenopausal; PM: postmenopausal; VAT: visceral adipose tissue; VAI: visceral adiposity index; LAP: lipid accumulation product; BMI: body mass index; TG: triglycerides; HDL-C: high-density lipoprotein cholesterol; IR: insulin resistance; HOMA-IR: homeostasis model assessment of insulin resistance; MetS: metabolic syndrome; DXA: dual-energy X-ray absorptiometry; BRI: body roundness index; AUC: area under the curve; OR: odds ratio; CI: confidence interval; MUH: metabolically unhealthy phenotype; MedDiet: Mediterranean diet; RCT: randomized controlled trial.

**Table 3:** Heart Rate Variability and Autonomic Function across the Menopausal Transition.

	Study Design		
Author [Ref.] Year	M-Stage/Control Sample Size/ Country	Design Intervention/Length	Main Outcomes
Sundas et al. [52] 2025 (REV)	General population *n* = 39 studies	Scoping Review HRV uncovered areas/No time limit	HRV significant in assessing ANS
Thakkar et al. [53] 2025 (REV)	PERI, PM, *n =* 100 India	Cross sectional. Aged 40–60 yrs 6 min. walk test (6MWT) HRV, BMI, aerobic capacity	HRV declines with advancing menopause. No significant correlations between HRV and 6MWT or BMI
De Jager et al. [54] 2026 (REV)	Menstrual cycle, hormonal stages *n* = 16 studies	Systematic Review Use of wearable devices for HRV No time limit.	HRV varies due to with hormonal fluctuations and declines with aging and in post-M
von Holzen et al. [55] 2016 (REV)	All M- stages No fixed sample.	Narrative review Endo- and exogenous estrogen effects on HRV.	HRV declines in PM. MHT partially restores HRV in PM
Carvalho et al. [56] 2022	PM *n* = 123 Brazil	Cross-Sectional HRV, Early vs. late PM groups	The increase in PM time decreases HRV indices
Ramesh et al. [57] 2022	PRE, PM. *n* = 41 Canada	Cross-sectional HRV, Estradiol levels recording	HRV declines primarily associated with age rather than estradiol.
Solanki et al. [58] 2025	PRE, PERI, PM *n* = 314 India	Cross-sectional HRV in 5 age-matched groups 40–55 yrs	No HRV differences after age adjustment; age is key determinant
Rezende-Barbosa et al. [59] 2017	PM *n* = 39 Brazil.	Interventional HRV, Functional training. 18 weeks	Functional training increases SDNN and α1, α1/α2 of DFA
Sakai et al. [60] 2020	PM *n* = 26 Japan	Interventional HRV, Autogenic training, Skin properties 7 weeks	Autogenic training improves ANS modulation
Putra et al. [61] 2024	PM *n* = 29 Indonesia	Interventional Exercise Training 2 weeks	Exercise increases SDNN
Praveena et al. [62] 2018	PM *n* = 67 India	Interventional HRV, yoga practice vs. no yoga practice 3 months	Yoga improves autonomic balance
Virtanen et al. [63] 2024	PERI, PM *n* = 35 Finland	Interventional HRV, Sleep study, hormone therapy 6 months	Sleep disturbance increased HRV in PM, no Hormone therapy effect on HRV
Virtanen et al. [64] 2015	PM, Young women *n* = 31 Finland	Interventional HRV, 40 h sleep deprivation	Sleep deprivation worsens ANS, especially in PM. Hormone therapy does not give protection
De Zambotti et al. [65] 2017	PERI *n* = 43 USA	Observational HRV, Menstrual stages Insomnia vs. control	PERIM with insomnia has increased HR during sleep, compared with age-matched controls
Sanchez-Barajas et al. [66] 2015	PERI, PM *n* = 100 México	Cross-Sectional ECG, hormones and carotid US indices.	Carotid indices are similar. Higher SDNN in post-M women.
Sanchez-Barajas et al. [67] 2018	PRE, PM *n* = 177 México	Cross-Sectional HRV, CIMT, FMD	CIMT has higher predictive value for early cardiovascular damage at PM. Worse vascular markers in late PM.
Jones et al. [68] 2015	PERI, PM *n* = 282 USA	Interventional HRV, VMS, physical activity, Omega 3 12 weeks	No association between HRV and VMS regardless of activity or M-Stage
Stokes et al. [69] 2025	PM *n* = 69 USA	Cross sectional With vs. without VMS HRV at rest and during stress tests	VMS group showed higher HRV (SDNN), and lower HR than non-VMS group
Martinelli et al. [70] 2020	MT, PM *n* = 109 Brazil	Cross-sectional. HRV compared across menopausal symptom severity stages.	HRV reduced with increasing menopausal symptom severity. Sympathetic predominance in severe group.
Sahu et al. [71] 2024	PRE, PM *n* = 140 India	Cross-sectional. HRV and menopausal symptoms 40–55 yrs	PM showed lower HRV than PRE. Menopausal symptoms negatively correlated with parasympathetic indices
Philbois et al. [72] 2024	PM No fixed sample. Brazil	Review/observational. HRV in hypertensive and normotensive PM	PM hypertensive women show further reduced HRV vs. normotensive PM.
Renna et al. [73] 2022	Breast cancer survivors not M-stage stratified. *n* = 178 USA	Longitudinal observational HRV measured at multiple time points during and after acute psychosocial stress. Single laboratory session with follow-up	Distress disorder history predicted blunted HRV recovery after stress; women with prior anxiety/depression showed sustained reduced HRV vs. controls
Nattero-Chávez et al. [74] 2023	PERI, PM *n* = 332 Spain	Cross-sectional. Type 1 diabetes Women and men <50 yrs, >50 yrs HRV, CAN and cardiac autonomic reflex tests. Sex steroids measured.	No overall sex difference in CAN prevalence. CAN risk increases in menopause (old women) but not in old men. Women showed more severe CAN than men. Androgens were positively associated with HRV in men and negatively in women.
Pervaiz et al. [75] 2023	PRE, PM *n* = 80 Pakistan	Observational. Type 2 diabetes HRV, QTc interval (CAN marker)	HRV difference non-significant among groups. Prolonged QTc was prevalent and associated with markers of autonomic dysfunction in PM diabetic women
Haldar et al. [76] 2026	PRE, PM *n* = 240 India	Cross-sectional HRV, cognitive function	PM showed lower cognitive scores and altered HRV compared to PRE. Positive association between HRV and cognition.
Duval et al. [77] 2025 [REV]	General female population HRV, cognition	Narrative review Menstrual cycle, menopause transition, Polycystic ovary syndrome	vmHRV and cognition show alterations in females across the adult lifespan. Few studies have directly addressed their interaction
Almeida et al. [78] 2021	PM *n* = 96 Brazil.	Cross-sectional. HRV, Dry eye syndrome (DES).	No association between DES and HRV. Clinical factors, time since menopause, and symptom intensity were not associated with HRV
Scatà et al. [79] 2024	PRE, PM *n* = 48 Brazil/Italy	Cross-sectional. Active standing test (supine-to-stand). HRV and hemodynamic monitoring	PM showed blunted cardiac autonomic response to standing (attenuated HRV modulation)
Kangas et al. [80] 2016	Men, PRE, PM) ~45 yrs. *n* = 334 Finland	Cross-sectional Healthy men and women, ~45 yrs. Hemodynamics and cardiac workload in supine and upright positions	Sex differences in hemodynamic, cardiac workload and autonomic response to posture
Tolunay et al. [81] 2022	PM, *n* = 130 Turkey	Cross-sectional 45–60 yrs aged HRV, sexual activity status. Menopausal symptoms	Sexual activity associated with more favorable autonomic profile
Voss et al. [82] 2015	KORA S4 data base Healthy subjects *n* = 1906 Germany	Cross-Sectional HRV, Aged groups of men vs. women 25–74 yrs	HRV gender differences disappear with age

Abbreviations: HRV, heart rate variability; ANS, autonomic nervous system; PRE, premenopausal; PERIM, perimenopausal; PM, postmenopausal; MT, menopausal transition; MHT, menopausal hormone therapy; VMS, vasomotor symptoms; SDNN, standard deviation of NN intervals; CAN, cardiac autonomic neuropathy.

**Table 4:** Exercise Interventions and Their Effects in Postmenopausal Women.

Exercise Domain	Exercise Type	Intervention	Duration and Frequency	Main Effects
Aerobic dance-based [115,116,117,118,140,141]	Moderate-intensity walking	Continuous aerobic training	30–60 min, 3–5 times/week, 12–24 weeks	Reduced BMI and vascular inflammatory markers; improved cardiometabolic profile
	Jazz dance	Structured dance sessions	60 min, 3 times/week, 16–24 weeks (6–12-month follow-up)	Improved cardiorespiratory fitness, body composition, muscle strength, and sleep quality
	Jazz dance & concurrent training	Combined modalities	3 times/week, 12–24 weeks	Reduced menopausal symptoms and improved quality of life
	Recreational team handball	Group-based sport	60 min, 2–3 times/week, 12–20 weeks	Improved aerobic fitness, cardiometabolic profile, balance, and body composition
	Aerobic exercise (osteopenic women)	Targeted aerobic training	45–60 min, 3 times/week, 12 weeks	Increased bone formation markers, reduced resorption markers, and improved quality of life
Resistance training [120,121,122,124,128,142,144]	Resistance band training	Elastic resistance	45–60 min, 3 times/week, 12–24 weeks	Improved muscle mass, blood pressure, hormonal profile, and metabolic syndrome risk
	Resistance training (different loads)	Progressive overload	2–3 times/week, 12–16 weeks	Reduced inflammatory biomarkers; improved muscle strength and physical performance
	Acute resistance exercise	Low vs. high load	Single session	Improved glucose tolerance in a load-dependent manner
	Resistance training (estrogen status)	Hormone-modulated training	3 times/week, 12 weeks	Estrogen status influenced metabolic adaptations
	Resistance training and supplementation	Fish oil or L-leucine	2–3 times/week, 12–24 weeks	Improved physical function, cardiometabolic health, and adipokine profile
Concurrent/combined training [130,131,143]	Aerobic and resistance training	Combined training	3 times/week, 12–16 weeks	Reduced fat mass, increased lean mass, and modulation of gut microbiota
	Concurrent vs. HIIT	Comparative intervention	3 times/week, 12 weeks	Improved physical function; HIIT showed greater time efficiency
	Aerobic-resistance and royal jelly	Supplemented combined training	3 times/week, 8–12 weeks	Increased antioxidant enzyme activity and improved liver function
High intensity/interval training [129,130]	Sprint interval training	Short high-intensity bouts	3 times/week, 12 weeks	Reduced total and visceral fat mass; increased lean mass
	High-intensity interval training (HIIT)	Interval training	20–30 min, 3 times/week, 12 weeks	Improved physical function and reduced fat mass
Bone-targeted/impact exercise [124,125,127,132,133,134,135]	Yi Jin Jing and resistance exercise	Combined traditional and resistance training	5 times/week, 12 months	Increased whole-body bone mineral density
	Whole-body vibration	Mechanical stimulation	2–3 times/week, 6–12 months	Improved muscle power; modest increases in bone mineral density
	Neuromuscular training	Functional protocols	2–3 times/week, 12–24 weeks	Increased regional bone mass
	High-impact exercise	Continuous or intermittent	3 times/week, 12 months	Increased lumbar spine and femoral neck bone mineral density
	Bone-loading exercise w/without medication	Exercise with/without pharmacological support	12 months	Reduced fracture risk; effects comparable to risedronate

Abbreviations: BMI, body mass index; HIIT, high-intensity interval training.

## Data Availability

No new data were created or analyzed in this study.
